# On the Approximated Reachability of a Class of Time-Varying Nonlinear Dynamic Systems Based on Their Linearized Behavior about the Equilibria: Applications to Epidemic Models

**DOI:** 10.3390/e21111045

**Published:** 2019-10-26

**Authors:** Manuel De la Sen

**Affiliations:** Institute of Research and Development of Processes IIDP, University of the Basque Country, Campus of Leioa, P.O. Box 48940, Leioa, 48940 Bizkaia, Spain; manuel.delasen@ehu.eus

**Keywords:** controllability, reachability, nonlinear dynamics, linearization, biological processes, epidemic models, entropy, self-organization, targeted state/output

## Abstract

This paper formulates the properties of point reachability and approximate point reachability of either a targeted state or output values in a general dynamic system which possess a linear time-varying dynamics with respect to a given reference nominal one and, eventually, an unknown structured nonlinear dynamics. Such a dynamics is upper-bounded by a function of the state and input. The results are obtained for the case when the time-invariant nominal dynamics is perfectly known while its time-varying deviations together with the nonlinear dynamics are not precisely known and also for the case when only the nonlinear dynamics is not precisely known. Either the controllability gramian of the nominal linearized system with constant linear parameterization or that of the current linearized system (which includes the time-varying linear dynamics) are assumed to be non-singular. Also, some further results are obtained for the case when the control input is eventually saturated and for the case when the controllability gramians of the linear parts are singular. Examples of the derived theoretical results for some epidemic models are also discussed.

## 1. Introduction

Usually, real dynamic systems are neither time-invariant nor linear in their whole operation rank since there are usually saturation and dead-zone type nonlinearities at the input, saturated behaviors in the state and output variables and sometimes nonlinear dynamics. See, for instance [1,2,3] and some references therein. However, very relevant information about their properties is often obtained from the knowledge of their equilibrium points, or their equilibrium steady-state oscillations, and the Jacobian matrices which describe the linearized trajectory solutions around such point for small deviations of linearity. This is the case, for instance, in some biological problems describing the species evolution through time [4] or in mathematically modelled epidemic models described by either differential, difference or hybrid equations. In particular, most of the epidemic models under current use and study possess at least one disease-free equilibrium point at which the infective subpopulations are null and an endemic one at which the infective subpopulations are non-null. A so-called reproduction number, which is calculated from the model parameters, establishes if the infectious asymptotically vanishes converging to an asymptotically stable disease-free attractor (if the reproduction number is less than unity) or it becomes endemic if such a number exceeds unity. Some of the relevant properties of positivity and stability of epidemic models are already qualitatively reflected in their linearized versions around their equilibrium points. See, for instance, [5,6] and references therein. The background literature on epidemic models is very abundant, including the use of either vaccination or treatment controls as well as combinations of both types of controls. See, for instance, [5,6,7,8,9,10,11,12,13,14] and the references therein. Such controls may reduce the value of the basic reproduction number related to the case of absence of controls, so the average number of contagions per each primary infectious case, and they are also able to change the components of the equilibrium points, that is the numbers of each subpopulation at the equilibrium, and the rates the convergence to such equilibrium points. The usual epidemic models are typically based on differential, difference or mixed equations which describe the coupled dynamics of the various subpopulation or, in general, they can include point and distributed delayed dynamics or to be also formulated in a stochastic framework, [7,8,11]. There are also studies for models of networks available which include different nodes which can represent different sets of interacting communities [14,15], which combined control strategies which take into account the communication links and population flows. Some of the models introduce appropriate either prediction or entropy tools or game theory to discuss the increase of disorder associated to them. See, for instance [11,12,13,14]. In particular, the entropy aspects are focused on deciding the various probabilities of different steady-state behaviors or to elucidate if the mathematical model is working properly, that is, if the entropy is non-negative [11].

The main objective of this paper is the study of the point reachability and point output-reachability and their approximate counterparts in the presence of uncertain dynamics, at a prescribed time instant, of either a targeted state or targeted output value in a dynamic system which has a linear time-varying dynamics with respect to a given nominal one and an unknown structured nonlinear dynamics with a known upper-bounding function. The results are given for the case when the time-invariant nominal dynamics is known while the time-varying deviations and the nonlinear dynamics are not precisely known and for the case when only the nonlinear dynamics is not precisely known. In the first case, the controllability gramian of the nominal linearized system with constant linear parameterization is assumed non-singular. In the second case, the current linearized system (which includes the time-varying linear dynamics) is assumed to be non-singular. Later on, some formal extensions are given for the case when the control input is saturated and for the case when the above mentioned controllability gramians are singular under certain ad hoc algebraic type constraints on the targeted state or targeted output. Some applications of the derived theoretical results for some epidemic models are also discussed. The paper content is organized as follows. Section 2 and Section 3 of this paper are concerned with the study of general dynamic system whose linear part is time-varying, formulated as a deviation from a constant nominal behaviour, and the nonlinear dynamics are introduced through unstructured functions which are, in general, dependent on the state and output. The state and output trajectory solutions are given analytically through closed formulas. Two key simplified auxiliary linear systems, which are linearized versions of the whole nonlinear system, are introduced and discussed, namely: (a) that describing the nominal linearized dynamics, in which the time-varying deviation of the linear dynamics respect to their nominal values and the nonlinear contributions are deleted; (b) that describing the linear time varying dynamics by neglecting the nonlinear contributions to the dynamics. The controllability and reachability properties of those auxiliary systems are formulated based on the corresponding controllability gramians. It is also examined and quantified to what extent the reachability of the whole nonlinear system is achievable in an approximate way provided that the linearized system versions are reachable. The (state) reachability is discussed at the levels of point-reachability (the targeted state in finite time is prefixed) or general reachability (the targeted state is arbitrarily fixed). The tolerances, in term of worst-case targeting errors related to a targeted state, of the approximate point-reachability of the whole nonlinear system are discussed provided that either the nominal or the current linearized systems are point-reachable. In particular, the analysis of Section 3 is performed on the whole nonlinear dynamics under the assumptions that the auxiliary linearized systems are point-reachable. It has to be pointed out that the controls used for approximate state targeting of the whole nonlinear system are those used for reachability of the linearized counterparts. Special attention is paid to the case when the norms of the nonlinear contributions to the dynamics are upper-bounded by weighted powers of the state and input norms. On the other hand, counterpart versions for output reachability are discussed in Section 4 as simple extensions based on the manipulations of the output controllability gramians of the auxiliary linearized systems. Section 4 pays also attention to the cases when the controllability gramian is rank-defective, so singular, and either there are non-unique solutions to the point-reachability problem (that is, the system is not controllable and the relevant algebraic system that formulates the problem is compatible indeterminate) or there are no algebraic solutions (that is, such an algebraic system is incompatible). In those cases, the alternative formulation is based on the use of Moore-Penrose pseudoinverses of the controllability gramian [15,16]. Finally, this section also considers the case when the input is saturated, as it is often the case in many real problems, so that the theoretical input to achieve point-reachability of the nominal linearized system has to be “ad hoc” modified. This situation is formally treated by incorporating an extra reachability error to the suited targeted state or output being caused by the deviation of the injected control input from linearity. Section 5 discusses some worked examples related to epidemic models in the contexts of stability and point-reachability based on the behaviors and related properties of their linearized versions. The controls are either vaccination efforts on the susceptible or antiviral or antibiotic treatment on the infectious both based on feedback information. On the other hand, some auxiliary results needed for the main ones are given in the appendixes. A simple discussion which might highlight the use of entropy in an information context of the relevance to the trajectories in the presence of more than one attractor is given in one of the given examples. The notation used in the following sections is $I_{n}$ is the n−th identity matrix, the superscript T stands for transposition and $\lambda_{\max}\left(.\right)$ and $\lambda_{\min}\left(.\right)$ are the maximum and minimum eigenvalues of the symmetric matrix (.).

## 2. Approximate Reachability of a Linear Time-Varying System under the Exact Reachability of Its Nominal Linearized Counterpart

Consider the single-input single-output linear time-varying dynamic system of order:

(1) $$\dot{x}\left(t\right)=A\left(t\right)x\left(t\right)+b\left(t\right)u\left(t\right)+\xi \left(x\left(t\right),u\left(t\right)\;,\;t\;\right)$$

(2) $$y\left(t\right)=c\left(t\right)x\left(t\right)+d\left(t\right)u\left(t\right)+\xi_{y}\left(x\left(t\right),u\left(t\right)\;,\;t\;\right)$$

subject to $x\left(0\right)=x_{0}$, which has non-linear unstructured contributions ξ(x(t),u(t) , t ) and $\xi_{y}\left(x\left(t\right),u\left(t\right)\;,\;t\;\right)$ to the state and output and with the linear part is given by (3)–(4), that is, the particular case of (1)–(2) with $\tilde{A}\left(t\right)=0$, $\tilde{b}\left(t\right)=0$, $\tilde{C}\left(t\right)=0$, $\tilde{D}\left(t\right)=0$,ξ(x(t),u(t) , t )=0 and $\xi_{y}\left(x\left(t\right),u\left(t\right)\;,\;t\;\right)=0$; $\forall t\in R_{0+}$), and:

(3) $$A\left(t\right)=A_{0}+\tilde{A}\left(t\right);\;b\left(t\right)=b_{0}+\tilde{b}\left(t\right)$$

(4) $$c\left(t\right)=c_{0}+\tilde{c}\left(t\right);\;d\left(t\right)=d_{0}+\tilde{d}\left(t\right)$$

where $x\left(t\right)\in R^{n}$ is the state vector, u(t)∈R and y(t)∈R are the scalar input and output, respectively, and the matrices of dynamics $A_{0}\;,\;A\left(t\right)\;,\;\tilde{A}\left(t\right)\in R^{n\times n}$; the control vectors $b\left(t\right)\;,\;b_{0\;},\;\tilde{b}\left(t\right)\in R^{n}$; and the output vectors $d\left(t\right)\;,\;d_{0\;},\;\tilde{d}\left(t\right)\in R^{p}$. Note that:

1) If $\tilde{A}\left(t\right)=0$, $\tilde{b}\left(t\right)=0$,$\tilde{c}\left(t\right)=0$,$\tilde{d}\left(t\right)=0$, ξ(x(t),u(t) , t )=0 and $\xi_{y}\left(x\left(t\right),u\left(t\right)\;,\;t\;\right)=0$; $\forall t\in R_{0+}$, then the resulting dynamic system (1)–(2), subject to (3)–(4), is said to be the “*nominal linearized system*”
2) The so-called “*current linearized system*” is distinct from the nominal linearized one and describes the situation when at least one of these parametrical perturbation matrices is not identically zero for all time while the nonlinear contributions are still identically zero for all time.
3) The complete system with all the effects in its dynamics including the time-varying parametrical disturbances of the matrices and nonlinear contributions is refereed to as the “*current system*”.

The system (1)–(2), subject to (3)–(4) can be rewritten as:

(5) $$\dot{x}\left(t\right)=A_{0}x\left(t\right)+b_{0}u\left(t\right)+v_{x0}\left(t\right);\;v_{x0}\left(t\right)=\tilde{A}\left(t\right)x\left(t\right)+\tilde{b}\left(t\right)u\left(t\right)+\xi \left(x\left(t\right),u\left(t\right)\;,\;t\;\right)$$

(6) $$y\left(t\right)=c_{0}^{T}x\left(t\right)+d_{0}u\left(t\right)+v_{y0}\left(t\right);\;v_{y0}\left(t\right)={\tilde{c}}^{T}\left(t\right)x\left(t\right)+\tilde{d}\left(t\right)u\left(t\right)+\xi_{y}\left(\left(x\left(t\right),u\left(t\right)\;,\;t\;\right)\right)$$

Now the approximate controllability of the current linear time-varying system (1)–(2), subject to (3)–(4) under the controllability of its nominal linearized time-invariant counterpart is discussed. Assume that for any prefixed time interval [0 , t], the control law is:

(7) $$u\left(\tau \right)=u_{0}\left(\tau \right)=b^{T}e^{A_{0}^{T}\left(t-\tau \right)}v_{0}\left(t\right);\;\tau \in \left[0\;,\;t\right]$$

where v(t) is an auxiliary control function:

(8) $$x\left(t\right)=e^{A_{0}t}x_{0}+\left(\int_{0}^{t}e^{A_{0}\left(t-\tau \right)}b_{0}b_{0}^{T}e^{A_{0}^{T}\left(t-\tau \right)}d\tau \;\right)v_{0}\left(t\right)+\int_{0}^{t}e^{A_{0}\left(t-\tau \right)}v_{x0}\left(\tau \right)d\tau$$

(9) $$=e^{A_{0}t}x_{0}+\left(\int_{0}^{t}e^{A_{0}\left(t-\tau \right)}b_{0}b_{0}^{T}e^{A_{0}^{T}\left(t-\tau \right)}d\tau \;\right)v_{0}\left(t\right)+\int_{0}^{t}e^{A_{0}\left(t-\tau \right)}\left(\tilde{A}\left(\tau \right)x\left(\tau \right)+\tilde{b}\left(\tau \right)u_{0}\left(\tau \right)\right)d\tau +\int_{0}^{t*}e^{A_{0}\left(t*-\tau \right)}\xi \left(x\left(\tau \right),u\left(\tau \right)\;,\;\tau \;\right)d\tau$$

under a initial condition $x\left(0\right)=x_{0}$, and:

(10) $$\begin{array}{c} y\left(t\right)=c_{0}^{T}\left[e^{A_{0}t}x_{0}+\left(\int_{0}^{t}e^{A_{0}\left(t-\tau \right)}b_{0}b_{0}^{T}e^{A_{0}^{T}\left(t-\tau \right)}d\tau \;\right)v_{0}\left(t\right)+\int_{0}^{t}e^{A_{0}\left(t-\tau \right)}v_{x0}\left(\tau \right)d\tau \right]+d_{0}u_{0}\left(t\right)+v_{y0}\left(t\right)\\ =c_{{}^{0}}^{T}\left[e^{A_{0}t}x_{0}+\left(\int_{0}^{t}e^{A_{0}\left(t-\tau \right)}b_{0}b_{0}^{T}e^{A_{0}^{T}\left(t-\tau \right)}d\tau \;\right)v_{0}\left(t\right)\right]+d_{0}u_{0}\left(t\right)+c_{0}^{T}\int_{0}^{t}e^{A_{0}\left(t-\tau \right)}v_{x0}\left(\tau \right)d\tau +v_{y0}\left(t\right)\\ =c_{{}^{0}}^{T}\left[e^{A_{0}t}x_{0}+\left(\int_{0}^{t}e^{A_{0}\left(t-\tau \right)}b_{0}b_{0}^{T}e^{A_{0}^{T}\left(t-\tau \right)}d\tau \;\right)v_{0}\left(t\right)+\int_{0}^{t}e^{A_{0}\left(t-\tau \right)}\left(\tilde{A}\left(\tau \right)x\left(\tau \right)+\tilde{b}\left(\tau \right)u_{0}\left(\tau \right)+\xi \left(x\left(\tau \right),u\left(\tau \right)\;,\;\tau \;\right)\right)d\tau \right]\\ +d_{0}u_{0}\left(t\right)+\xi_{y}\left(t,\;x\left(t\right)\right)+\tilde{d}\left(t\right)u_{0}\left(t\right)\\ +{\tilde{c}}^{T}\left(t\right)\left[e^{A_{0}t}x_{0}+\left(\int_{0}^{t}e^{A_{0}\left(t-\tau \right)}b_{0}b_{0}^{T}e^{A_{0}^{T}\left(t-\tau \right)}d\tau \;\right)v_{0}\left(t\right)+\int_{0}^{t}e^{A_{0}\left(t-\tau \right)}b_{0}\left(\tilde{A}\left(\tau \right)x\left(\tau \right)+\tilde{b}\left(\tau \right)u_{0}\left(\tau \right)+\xi \left(x\left(\tau \right),u\left(\tau \right)\;,\;\tau \;\right)\right)d\tau \right] \end{array}$$

The following result relies on the approximate controllability of the current system under parametrical disturbances related to the nominal linearized one, assumed to be controllable.

**Theorem** **1.** *Assume that*$\left(A_{0}\;,\;b_{0}\right)$*is a controllable pair and that*$x^{*}\in R^{n}$*is any prefixed state value at an arbitrary given time*$t=t^{*}\in R_{+}$. *Then:*

(11) $$x\left(t^{*}\right)=F\left(t^{*}\right){}^{-1}\left[x^{*}+G\left(t^{*}\right)\left(x*-e^{A_{0}t*}x_{0}\right)\right]\;+\int_{0}^{t*}e^{A_{0}\left(t*-\tau \right)}\xi \left(x\left(\tau \right),u\left(\tau \right)\;,\;\tau \;\right)d\tau$$

*is reached for any initial condition*$x\left(0\right)=x_{0}$*under the control law:*

(12) $$u_{0}\left(\tau \right)=b_{0}^{T}e^{A_{0}^{T}\left(t^{*}-\tau \right)}v_{0}\left(t^{*}\right)=b_{0}^{T}e^{A_{0}^{T}\left(t^{*}-\tau \right)}G_{c\left[0,t*\right]}^{-1}\left(A_{0}\;,b_{0}\right)\left(x^{*}-e^{A_{0}t*}x_{0}\right);\;\tau \in {\left[0\;,\;t^{*}\right]}^{}$$

*via the auxiliary control function*$v_{0}\left(t*\right)=G_{c\left[0,t*\right]}^{-1}\left(A_{0}\;,b_{0}\right)\left(x^{*}-e^{A_{0}t*}x_{0}\right)$*, where:*

(13) $$G_{c0\left[0,t*\right]}\left(A_{0}\;,b_{0}\right)=\int_{0}^{t*}e^{A_{0}\left(t*-\tau \right)}b_{0}b_{0}^{T}e^{A_{0}^{T}\left(t*-\tau \right)}d\tau$$

is the controllability gramian of the nominal linearized system (that is, that associated with the controllable pair $\left(A_{0}\;,b_{0}\right)$*on*${\left[0,t^{*}\right]}^{}$), then non-singular, and:

(14) $$F\left(t*\right)=I_{n}-\int_{0}^{t*}e^{A_{0}\left(t*-\tau \right)}\tilde{A}\left(\tau \right)\Psi \left(\tau ,t*\right)d\tau$$

(15) $$G\left(t*\right)=\left(\int_{0}^{t*}e^{A_{0}\left(t*-\tau \right)}\tilde{b}\left(\tau \right)b_{0}^{T}e^{A_{0}^{T}\left(t*-\tau \right)}d\tau -\int_{0}^{t*}\int_{0}^{t*}e^{A_{0}\left(t*-\tau \right)}\tilde{A}\left(\tau \right)\Psi \left(\tau ,\sigma \right)b\left(\sigma \right)b_{0}^{T}e^{A_{0}^{T}\left(t*-\sigma \right)}d\sigma d\tau \right)G_{c\left[0,t*\right]}^{-1}\left(A_{0}\;,b_{0}\right)$$

*provided that*F(t*)*is non-singular,where*Ψ(t,τ)*is the fundamental matrix associated with*A(t)*so that*$\dot{\Psi }\left(t,\tau \right)=A\left(t\right)\Psi \left(t,\tau \right)$*;*$\forall t\left(\geq \tau \right),\tau \in R_{0+}$*, and:*

(16) $$\Psi \left(t,\tau \right)=e^{A_{0}\left(t-\tau \right)}+\int_{\tau }^{t}e^{A_{0}\left(t-\sigma \right)}\tilde{A}\left(\sigma \right)d\sigma ;\forall t\left(\geq \tau \right),\tau \in R_{0+}$$

**Proof.**  It turns out that controllability gramian of the pair $\left(A_{0}\;,b_{0}\right)$ is non-singular on ${\left[0,t^{*}\right]}^{}$ since the pair $\left(A_{0}\;,\;b_{0}\right)$ is controllable. Then, one gets from (9) and (12) that:

$$x\left(t\right)-e^{A_{0}t*}x_{0}-\int_{0}^{t*}e^{A_{0}\left(t*-\tau \right)}v_{x0}\left(\tau \right)d\tau =G_{c0\left[0,t*\right]}\left(A_{0}\;,b_{0}\right)v_{0}\left(t^{*}\right)$$

Then:

(17) $$\begin{array}{l} x\left(t^{*}\right)=x^{*}+\int_{0}^{t*}e^{A_{0}\left(t*-\tau \right)}\tilde{A}\left(\tau \right)x\left(\tau \right)d\tau +\int_{0}^{t*}e^{A_{0}\left(t*-\tau \right)}\left(\tilde{b}\left(\tau \right)u_{0}\left(\tau \right)+\xi \left(x\left(\tau \right),u\left(\tau \right)\;,\;\tau \;\right)\right)d\tau \\ ={\left[I_{n}+\int_{0}^{t*}e^{A_{0}\left(t*-\tau \right)}\tilde{b}\left(\tau \right)b_{0}^{T}e^{A_{0}^{T}\left(t*-\tau \right)}G_{c0\left[0,t*\right]}^{-1}\left(A_{0}\;,b_{0}\right)d\tau \right]}^{}x^{*}+\int_{0}^{t*}e^{A_{0}\left(t*-\tau \right)}\xi \left(x\left(\tau \right),u\left(\tau \right)\;,\;\tau \;\right)d\tau \\ +\int_{0}^{t*}e^{A_{0}\left(t*-\tau \right)}\tilde{A}\left(\tau \right)x\left(\tau \right)d\tau -\left(\int_{0}^{t*}e^{A_{0}\left(t*-\tau \right)}\tilde{b}\left(\tau \right)b_{0}^{T}e^{A_{0}^{T}\left(t*-\tau \right)}d\tau \right)\;G_{c0\left[0,t*\right]}^{-1}\left(A_{0}\;,b_{0}\right)e^{A_{0}t*}x_{0} \end{array}$$

with $x\left(0\right)=x_{0}$ and (16) holds from $\dot{\Psi }\left(t,\tau \right)=A\left(t\right)\Psi \left(t,\tau \right)$; $\forall t\left(\geq \tau \right),\tau \in R_{0+}$, since $A\left(t\right)=A_{0}+\tilde{A}\left(t\right)$ and $\Psi \left(t,t\right)=I_{n}$; $\forall t\in R_{0+}$. Then, one has:

(18) $$\Psi \left(\tau ,t\right)x\left(t\right)=\;x\left(\tau \right)+\int_{0}^{t}\Psi \left(\tau ,t\right)\Psi \left(t,\sigma \right)b\left(\sigma \right)u_{0}\left(\sigma \right)d\sigma =\;x\left(\tau \right)+\int_{0}^{t}\Psi \left(\tau ,\sigma \right)b\left(\sigma \right)u_{0}\left(\sigma \right)d\sigma ;\;\;\forall t\left(\geq \tau \right),\tau \in R_{0+}$$

Then, one gets, after replacing x(τ) from (18) into (17), that:

(19) $$x\left(t*\right)={\left[I_{n}+\int_{0}^{t*}e^{A_{0}\left(t*-\tau \right)}\tilde{b}\left(\tau \right)b_{0}^{T}e^{A_{0}^{T}\left(t*-\tau \right)}G_{c0\left[0,t*\right]}^{-1}\left(A_{0}\;,b_{0}\right)d\tau \right]}^{}x^{*}+\int_{0}^{t*}e^{A_{0}\left(t*-\tau \right)}\xi \left(x\left(\tau \right),u\left(\tau \right)\;,\;\tau \;\right)d\tau \;+\int_{0}^{t*}e^{A_{0}\left(t*-\tau \right)}\tilde{A}\left(\tau \right)\left(\Psi \left(\tau ,t^{*}\right)x\left(t*\right)-\int_{0}^{t*}\Psi \left(\tau ,\sigma \right)b\left(\sigma \right)u_{0}\left(\sigma \right)d\sigma \right)d\tau \;-\left(\int_{0}^{t*}e^{A_{0}\left(t*-\tau \right)}\tilde{b}\left(\tau \right)b_{0}^{T}e^{A_{0}^{T}\left(t*-\tau \right)}d\tau \right)\;G_{c0\left[0,t*\right]}^{-1}\left(A_{0}\;,b_{0}\right)e^{A_{0}t*}x_{0}$$

so that, after replacing the control law (12) on [0 , t*] into (19), one gets that:

(20) $${\left[\;I_{n}-\int_{0}^{t*}e^{A_{0}\left(t*-\tau \right)}\tilde{A}\left(\tau \right)\Psi \left(\tau ,t*\right)d\tau \right]}^{}x\left(t*\right)\;={\left[I_{n}+\int_{0}^{t*}e^{A_{0}\left(t*-\tau \right)}\tilde{b}\left(\tau \right)b_{0}^{T}e^{A_{0}^{T}\left(t*-\tau \right)}G_{c0\left[0,t*\right]}^{-1}\left(A_{0}\;,b_{0}\right)d\tau \right]}^{}x^{*}-\int_{0}^{t*}\int_{0}^{t*}e^{A_{0}\left(t*-\tau \right)}\tilde{A}\left(\tau \right)\Psi \left(\tau ,\sigma \right)b\left(\sigma \right)u_{0}\left(\sigma \right)d\sigma d\tau \;-\left(\int_{0}^{t*}e^{A_{0}\left(t*-\tau \right)}\tilde{b}\left(\tau \right)b_{0}^{T}e^{A_{0}^{T}\left(t*-\tau \right)}d\tau \right)\;G_{c0\left[0,t*\right]}^{-1}\left(A_{0}\;,b_{0}\right)e^{A_{0}t*}x_{0}+\int_{0}^{t*}e^{A_{0}\left(t*-\tau \right)}\xi \left(x\left(\tau \right),u\left(\tau \right)\;,\;\tau \;\right)d\tau \;={\left[I_{n}+\int_{0}^{t*}e^{A_{0}\left(t*-\tau \right)}\tilde{b}\left(\tau \right)b_{0}^{T}e^{A_{0}^{T}\left(t*-\tau \right)}G_{c0\left[0,t*\right]}^{-1}\left(A_{0}\;,b_{0}\right)d\tau \right]}^{}x^{*}\;-\left(\int_{0}^{t*}\int_{0}^{t*}e^{A_{0}\left(t*-\tau \right)}\tilde{A}\left(\tau \right)\Psi \left(\tau ,\sigma \right)b\left(\sigma \right)b_{0}^{T}e^{A_{0}^{T}\left(t*-\sigma \right)}d\sigma d\tau \right)\;G_{c0\left[0,t*\right]}^{-1}\left(A_{0}\;,b_{0}\right)\left(x^{*}-e^{A_{0}t*}x_{0}\right)\;-\left(\int_{0}^{t*}e^{A_{0}\left(t*-\tau \right)}\tilde{b}\left(\tau \right)b_{0}^{T}e^{A_{0}^{T}\left(t*-\tau \right)}d\tau \right)\;G_{c0\left[0,t*\right]}^{-1}\left(A_{0}\;,b_{0}\right)e^{A_{0}t*}x_{0}+\int_{0}^{t*}e^{A_{0}\left(t*-\tau \right)}\xi \left(x\left(\tau \right),u\left(\tau \right)\;,\;\tau \;\right)d\tau \;=x^{*}+\left[\int_{0}^{t*}e^{A_{0}\left(t*-\tau \right)}\tilde{b}\left(\tau \right)b_{0}^{T}e^{A_{0}^{T}\left(t*-\tau \right)}d\tau \;-\int_{0}^{t*}\int_{0}^{t*}e^{A_{0}\left(t*-\tau \right)}\tilde{A}\left(\tau \right)\Psi \left(\tau ,\sigma \right)b\left(\sigma \right)b_{0}^{T}e^{A_{0}^{T}\left(t*-\sigma \right)}d\sigma d\tau \right]\;G_{c0\left[0,t*\right]}^{-1}\left(A_{0}\;,b_{0}\right)\;\times \left(x*-e^{A_{0}t*}x_{0}\right)+\int_{0}^{t*}e^{A_{0}\left(t*-\tau \right)}\xi \left(x\left(\tau \right),u\left(\tau \right)\;,\;\tau \;\right)d\tau \;=x*+G\left(t*\right)\left(x*-e^{A_{0}t*}x_{0}\right)+\int_{0}^{t*}e^{A_{0}\left(t*-\tau \right)}\xi \left(x\left(\tau \right),u\left(\tau \right)\;,\;\tau \;\right)d\tau$$

Then (11) holds, subject to (13)–(15), if F(t*) is non-singular. □

**Definition** **1.** *The system (1)–(2), subject to (3)–(4), is said to be*$\left(x*,t^{*}\right)$*-point reachable from a given initial state*$x\left(0\right)=x_{0}$*(*$\mathrm{PR}\;\left(x^{*},t^{*}\;,x_{0}\right)$*) if there exists some control law*$u:{\left[0\;,\;t^{*}\right]}^{}\to R$*leading to the state targeting*$x\left(t^{*}\right)=x^{*}$for$t^{*}>0$*if*$x\left(0\right)=x_{0}$.

**Definition** **2.** *The system (1)–(2), subject to (3)–(4), is said to reachable*R*if it is*$\mathrm{PR}\;\left(x^{*},t^{*}\;,x_{0}\right)$*for any given triple*$\left(x_{0}\;,\;x^{*},\;t^{*}\right)$*. It is said to be reachable at time*$t^{*}$for an initial state$x\left(0\right)=x_{0}$*, say*$R\;\left(t^{*},\;x_{0}\right)$*, if it is*$\mathrm{PR}\;\left(x^{*},t^{*}\;,x_{0}\right)$*for any*$x^{*}$.

**Definition** **3.** *The control law (12) is said to be the reachability standard nominal control law (*$\mathrm{RSNCL}\left(x^{*},t^{*}\;,x_{0}\right))$*of (1)–(2), subject to (3)–(4), for the system to be*$\mathrm{PR}\;\left(x^{*},t^{*}\;,x_{0}\right)$.

**Remark** **1.**

1) *Note that the constraint (4) is irrelevant for (state)-reachability since the output is not specifically involved in such a property. However, we refer to that constraint in Definitions 1–3 to keep the whole system referred to fully defined through (1)–(4).*
2) *Note that if the parametrical disturbances of (3) are zeroed, so that the control and dynamics matrices are constant, then the resulting time-invariant linear system (that is, the nominal linearized one) is reachable, equivalently*$\mathrm{PR}\;\left(x^{*},t^{*}\;,x_{0}\right)$*for any given triple*$\left(x_{0}\;,\;x^{*},\;t^{*}\right)$*, under the*$\mathrm{RSNCL}\left(x^{*},t^{*}\;,x_{0}\right)$*, if and only if*$\left(A_{0}\;,b_{0}\right)$*is a controllable pair, equivalently if and only if its associate controllability gramian (13) is non-singular [1]. In particular, an existing control law which allows the targeting*$x\left(t^{*}\right)=x^{*}$for any given$x^{*}$at any given$t^{*}>0$*of the nominal linearized system from any given initial state*$x\left(0\right)=x_{0}$*is the*$\mathrm{RSNCL}\left(x^{*},t^{*}\;,x_{0}\right)$.

The following direct result relies on the eventual maintenance or lost of the reachability of the current linearized system related to the nominal linearized one if the parametrical disturbances are arbitrary. In particular, it is seen that, in general, the $\mathrm{RSNCL}\left(x^{*},t^{*}\;,x_{0}\right)$ does not allow exact prefixed state tracking even if the nominal linearized system is reachable.

**Theorem** **2.** *The following identity holds:*

(21) $$x\left(t^{*}\right)=\left[F\left(t^{*}\right){}^{-1}\left(\;I_{n}+G\left(t^{*}\right)\;\right)-I_{n}\right]\;x^{*}-F\left(t^{*}\right)G\left(t^{*}\right)e^{A_{0}t*}x_{0}+\int_{0}^{t*}e^{A_{0}\left(t*-\tau \right)}\xi \left(x\left(t\right),u\left(t\right)\;,\;t\;\right)d\tau$$

*the system (1)–(2), subject to (3)–(4) is* $\mathrm{PR}\;\left(x^{*},t^{*}\;,x_{0}\right)$ *under the* $\mathrm{RSNCL}\left(x^{*},t^{*}\;,x_{0}\right)$ *if some of the conditions given below holds:*

*(1)*  $\tilde{A}\left(t\right)\equiv 0$ *,* $\tilde{b}\left(t\right)\equiv 0$ *and* ξ(t ,x(t))≡0 *for* t∈[0, t*) *if* $\left(A_{0}\;,b_{0}\right)$ *is a controllable pair.*
*(2)*  x*=0*and*t*=+∞.
*(3)*  $x_{0}=0$ *and the controllability gramian of the nominal linearized parameterization on* ${\left[0\;,\;t^{*}\right]}^{}$ *is non-singular (that is, the pair* $\left(A_{0}\;,\;b_{0}\right)$ *is controllable) and it satisfies:*
  (22) $$G_{c0\left[0,t*\right]}\left(A_{0}\;,b_{0}\right)=\int_{0}^{t*}e^{A_{0}\left(t*-\tau \right)}b_{0}b_{0}^{T}e^{A_{0}^{T}\left(t*-\tau \right)}d\tau =\left(\int_{0}^{t*}e^{A_{0}\left(t*-\tau \right)}\tilde{A}\left(\tau \right)\Psi \left(\tau ,t*\right)d\tau \right){}^{-1}\;\times \left(\int_{0}^{t*}\int_{0}^{t*}e^{A_{0}\left(t*-\tau \right)}\tilde{A}\left(\tau \right)\Psi \left(\tau ,\sigma \right)b\left(\sigma \right)b_{0}^{T}e^{A_{0}^{T}\left(t*-\sigma \right)}d\sigma d\tau -\int_{0}^{t*}e^{A_{0}\left(t*-\tau \right)}\tilde{b}\left(\tau \right)b_{0}^{T}e^{A_{0}^{T}\left(t*-\tau \right)}d\tau \right)$$

**Proof.**  Equation (21) follows directly from (11) under the conditions (1)–(2). Condition (3) follows since one gets from (14)–(15) that $x_{0}=0$ implies that $x\left(t^{*}\right)=x*\neq 0$ if:

(23) $$I_{n}=F\left(t*\right)-G\left(t^{*}\right)=I_{n}-\int_{0}^{t*}e^{A_{0}\left(t*-\tau \right)}\tilde{A}\left(\tau \right)\Psi \left(\tau ,t*\right)d\tau \;+\left(\int_{0}^{t*}\int_{0}^{t*}e^{A_{0}\left(t*-\tau \right)}\tilde{A}\left(\tau \right)\Psi \left(\tau ,\sigma \right)b\left(\sigma \right)b_{0}^{T}e^{A_{0}^{T}\left(t*-\sigma \right)}d\sigma d\tau -\int_{0}^{t*}e^{A_{0}\left(t*-\tau \right)}\tilde{b}\left(\tau \right)b_{0}^{T}e^{A_{0}^{T}\left(t*-\tau \right)}d\tau \right)G_{c\left[0,t*\right]}^{-1}\left(A_{0}\;,b_{0}\right)$$

so that:

(24) $$\begin{array}{r} G_{c0\left[0,t*\right]}\left(A_{0}\;,b_{0}\right)=\int_{0}^{t*}e^{A_{0}\left(t*-\tau \right)}b_{0}b_{0}^{T}e^{A_{0}^{T}\left(t*-\tau \right)}d\tau =\left(\int_{0}^{t*}e^{A_{0}\left(t*-\tau \right)}\tilde{A}\left(\tau \right)\Psi \left(\tau ,t*\right)d\tau \right){}^{-1}\\ \times \left(\int_{0}^{t*}\int_{0}^{t*}e^{A_{0}\left(t*-\tau \right)}\tilde{A}\left(\tau \right)\Psi \left(\tau ,\sigma \right)b\left(\sigma \right)b_{0}^{T}e^{A_{0}^{T}\left(t*-\sigma \right)}d\sigma d\tau -\int_{0}^{t*}e^{A_{0}\left(t*-\tau \right)}\tilde{b}\left(\tau \right)b_{0}^{T}e^{A_{0}^{T}\left(t*-\tau \right)}d\tau \right) \end{array}$$

□

It turns out that this last constraint is generically unfeasible for almost any parametrical disturbances $\left(\tilde{A}\left(\tau \right)\;,\;\tilde{b}\left(\tau \right)\right)$ for τ∈[0 , t*]. As a result, we conclude that the exact reachability is achievable in the nominal linearized case, that is, in the absence of parametrical disturbances of the dynamics and control vector for some finite time t* if the controllability gramian is nonsingular for any interval ${\left[0\;,\;t_{1}^{*}\right)}^{}$ and some $t_{1}^{*}\in \left(0,\;t^{*}\right)$. Also, it turns out that this property holds for any finite t*>0 if and only if the pair $\left(A_{0}\;,\;b_{0}\right)$ is a controllable pair because of the formal analytical relation of the controllability gramian with the controllability matrix associated with the pair $\left(A_{0}\;,\;b_{0}\right)$. If the controllability gramian is nonsingular then point reachability is not generically achievable under arbitrary parametrical disturbances for any given initial condition $x_{0}$ and any targeted state x* at any finite time t*>0.

**Remark** **2.** *Note from (11) that:*

(25) $$\left\|F\left(t*\right){}^{-1}\right\|\;\;\left|\max\left[\;\left(1+\left\|G\left(t*\right)\right\|\right)\;\left\|x^{*}\right\|-\left\|G\left(t*\right)\right\|\left\|e^{A_{0}t*}\right\|\left\|x_{0}\right\|\;,\;\;\left\|x^{*}\right\|-\left\|G\left(t*\right)\right\|\left(\left\|x^{*}\right\|+\left\|e^{A_{0}t*}\right\|\left\|x_{0}\right\|\right)\right]\right|\;\leq \left\|x\left(t*\right)\right\|\leq \left\|F\left(t*\right){}^{-1}\right\|\;\left[\left(1+\left\|G\left(t*\right)\right\|\right)\;\left\|x^{*}\right\|+\left\|G\left(t*\right)\right\|\left\|e^{A_{0}t*}\right\|\left\|x_{0}\right\|\;\right]$$

If (A11) in Lemma A2 of Appendix A holds for t = t* *and the conditions 1–5 of Lemma A4 hold then:*

(26) $$\left\|G\left(t*\right)\right\|\leq (\frac{K_{0}^{2}}{2\rho_{0}}\;\left(1-e^{-2\rho_{0}t*}\right)\left\|b_{0}\right\|\varepsilon_{b}^{*}+K_{0}^{2}K_{\Psi }\;\left\|b_{0}\right\|\varepsilon_{b}^{*}\varepsilon_{A}^{*}\;\times e^{-2\rho_{0}t*}\frac{e^{-2\rho_{\Psi }t*}+1-e^{\left(\rho_{0}-\rho_{\Psi }\right)t*}-e^{-\left(\rho_{0}+\rho_{\Psi }\right)t*}}{\rho_{0}^{2}-\rho_{\Psi }^{2}})\;\left\|G_{c\left[0,t*\right]}^{-1}\left(A_{0}\;,b_{0}\right)\right\|$$

*Then, from Lemmas A2–A4 and defining* $\varepsilon_{\xi }^{*}=\varepsilon_{\xi }\left(t^{*},u_{0}\left[0\;,\;t^{*}\right)\right)=\frac{K_{0}}{\rho_{0}}\;\underset{0\leq \tau \leq t^{*}}{\sup}\left\|\xi \left(\tau ,\;x\left(\tau \right)\right)\right\|$ *, one has:*

(27) $$\varepsilon_{iFm}\left(t^{*}\right)\;\;(\left\|x^{*}\right\|-\varepsilon_{G}^{*}\left(\left\|x*\right\|+\left\|e^{A_{0}t*}\right\|\left\|x_{0}\right\|\right))-\varepsilon_{\xi }^{*}\left(1-e^{-\rho_{0}t^{*}}\right)\;\leq \left\|x\left(t*\right)\right\|\leq \varepsilon_{iFM}\left(t^{*}\right)\;\;(\left\|x^{*}\right\|+\varepsilon_{G}^{*}\left(\left\|x*\right\|+\left\|e^{A_{0}t*}\right\|\left\|x_{0}\right\|\right))+\varepsilon_{\xi }^{*}\left(1-e^{-\rho_{0}t^{*}}\right)$$

*where* $\varepsilon_{G}^{*}$ *is defined in Lemma A4 (iii),* $\varepsilon_{iFM}^{*}=\varepsilon_{iFM}\left(t^{*}\right)$ *and* $\varepsilon_{iFm}^{*}=\varepsilon_{iFm}\left(t^{*}\right)$ *are defined for* t=t* *and:*

*a)*  *for* i=5 *in (A12) and (A16) if (A11) (i.e., the assumptions 5 of Lemma A2) holds,*
*b)*  *for* i=4 *in (A13) and (A17) if (A10) (i.e., the assumption 4 of Lemma A2) holds,*
*c)*  *for* i=3 *in (A14) and (A18) if (A9) (i.e., the assumption 3 of Lemma A2) holds,*
*d)*  *for* i=2 *in (A15) and (A19) if (A8) (i.e., the assumption 2 of Lemma A2) holds,*

*respectively,*

*Note by inspecting Lemmas A2–A4 that* $\varepsilon_{G}^{*}=1+o\left(\max\left(\varepsilon_{A}^{*}\;,\;\varepsilon_{b}^{*}\right)\;\left|1-e^{-\rho_{0}t*}\right|\right)$ *, and* $\varepsilon_{iFM}^{*}=1+o\left(\varepsilon_{A}^{*}\left|1-e^{-\rho_{0}t*}\right|\right)$ *,* $\varepsilon_{iFm}^{*}=1-o\left(\varepsilon_{A}^{*}\left|1-e^{-\rho_{0}t*}\right|\right)$ *under the corresponding condition for* i=2,3,4,5 *among the above set of conditions. Then:*

(28) $$\left\|x\left(t^{*}\right)\right\|\in \left[\left|1-o\left(\max\left(\varepsilon_{A}^{*}\;,\;\varepsilon_{b}^{*},\varepsilon_{\xi }^{*}\right)\;\left|1-e^{-\rho_{0}t*}\right|\right)\right|\;\left\|x^{*}\right\|,\;\left(1+o\left(\max\left(\varepsilon_{A}^{*}\;,\;\varepsilon_{b}^{*},\varepsilon_{\xi }^{*}\right)\;\left|1-e^{-\rho_{0}t*}\right|\right)\right)\left\|x^{*}\right\|\right]$$

*The stability of the open-lop nominal linearized system is not crucial in the above results. If*$\rho_{0}\leq 0$*(i.e., the critically stable and unstable cases for the nominal linearized system), then*$\left(-\rho_{0}\right)\to \left|\rho_{0}\right|$*and*$\left(1-e^{-\rho_{0}t{}^{*}}\right)\to \left|e^{\left|\rho_{0}\right|t^{*}}-1\right|$*in all the relevant equations in the main body and Lemmas A2 and A4 in the Appendixes to get alternative results for those cases. So, Equation (28) applies for any absolute value and sign of the stability abscissa of the matrix*$A_{0}$.

*Note that (27)–(28) lead to a worst-case targeting state estimate at time* $t^{*}$ *through the control law (12) if the nominal linearized system is controllable. The controllability of the nominal linearized system translates into an approximate parallel result of approximate reachability of the whole current system and the approximation degree increases, as expected, as the parametrical disturbances and the sizes of the nonlinear contributions decrease. Thus, we have the following “ad hoc” definition and theorem concerning this issue.*

**Definition** **4.** *The current system (1)–(2), subject to (3)–(4), is said to be* $\left(x*,t^{*}\right)$ *-point* (1−α) *-approximately reachable* ${\mathrm{APR}}_{1-\alpha }\;\left(x^{*},t^{*}\;,x_{0}\right)$ *, with* $\alpha =o\left(\max\left(\varepsilon_{A}^{*}\;,\;\varepsilon_{b}^{*},\varepsilon_{\xi }^{*}\right)\;\left|e^{\left|\rho_{0}\right|t^{*}}-1\right|\right)$ *, from a given initial state* $x\left(0\right)=x_{0}$ *, where:*

$\varepsilon_{\xi }^{*}=\varepsilon_{\xi }\left(t^{*},u_{0}\left[0\;,\;t^{*}\right)\right)=\frac{K_{0}}{\rho_{0}}\;\underset{0\leq \tau \leq t^{*}}{\sup}\left\|\xi \left(\tau ,\;x\left(\tau \right)\right)\right\|$*if there exists some control law*$u:{\left[0\;,\;t^{*}\right]}^{}\to R$*leading to the state targeting*$x\left(t^{*}\right)\in {\left[\left(1-\alpha \right)x^{*},\left(1+\alpha \right)x^{*}\;\right]}^{}$*for*$t^{*}>0$*if*$x\left(0\right)=x_{0}$.

*Since* $x\left(t^{*}\right)=x^{*}$ *if* α=0 *then one has:*

**Assertion** **1.** *The current system (1)–(2), subject to (3)–(4), isif and only if it is*$\mathrm{PR}\;\left(x^{*},t^{*}\;,x_{0}\right)$.

The above considerations, together with Remark 2 and Lemmas A2 and A4 in Appendix A, lead to the following direct result concerning the reachability of the current system if the nominal linearized one is asymptotically stable and controllable:

**Theorem** **3.** *(approximate reachability of the current system). Assume that:*

*1)*  $A_{0}$*is a stability matrix with stability abscissa*$\left(-\rho_{0}\right)<0$*so that*$\left\|e^{A_{0}t}\right\|\leq K_{0}e^{-\rho_{0}t}$*;*$\forall t\in R_{0+}$*for some real constant*$K_{0}\left(\geq 1\right)$,
*2)*  *The pair* $\left(A_{0},\;b_{0}\right)$ *is controllable, that is, it is* $\mathrm{PR}\;\left(x^{*},t^{*}\;,x_{0}\right)$ *for any given triple* $\left(x_{0}\;,\;x^{*},\;t^{*}\right)$ *so reachable,*
*3)*  $\left\|\int_{0}^{t}e^{A_{0}\left(t-\tau \right)}\tilde{A}\left(\tau \right)\Psi \left(\tau ,t\right)d\tau \right\|<1$ *(guaranteed via the sufficient conditions (A9)- (A11) [Lemma A2 of Appendix A],*
*4)*  $\left\|\Psi \left(t,\tau \right)\right\|\leq K_{\Psi }e^{-\rho_{\Psi }\left(t-\tau \right)}$ *;* ∀t(≥τ), τ∈R *with* $K_{\Psi }\geq 1$ *and* $0<\rho_{\Psi }<\rho_{0}$

*Then, the current linearized system (1)–(2), subject to (3)–(4) (i.e., that resulting for* ξ(x(t),u(t) , t )≡0 *on* ${\left[0\;,\;t^{*}\right]}^{}$ *), is exponentially stable. Furthermore, the whole system (1)–(2), subject to (3)–(4), is* ${\mathrm{APR}}_{1-\alpha }\;\left(x^{*},t^{*}\;,x_{0}\right)$ *with degree* $\alpha =o\left(\max\left(\varepsilon_{A}^{*}\;,\;\varepsilon_{b}^{*},\varepsilon_{\xi }^{*}\right)\;\left|\;1-e^{-\rho_{0}t*}\right|\right)$ *, from a given initial state* $x\left(0\right)=x_{0}$ *under the nominal control law* $u_{0}:{\left[0\;,\;t^{*}\right]}^{}\to R$ *of Equation (12), where:*

$\underset{0\leq t<\infty }{\sup}\left\|\tilde{A}\left(t\right)\right\|\leq \varepsilon_{A}^{*}$ *;* $\underset{0\leq t<\infty }{\sup}\left\|\tilde{b}\left(t\right)\right\|\leq \varepsilon_{b}^{*}$ *;* $\varepsilon_{\xi }^{*}=\frac{K_{0}}{\rho_{0}}\;\underset{0\leq \tau \leq t^{*}}{\sup}\left\|\xi \left(x\left(\tau \right),u\left(\tau \right)\;,\;\tau \;\right)\right\|$

**Theorem** **4.** *Assume that the current linearized system (1)–(2) subject to (3)–(4), is* $\mathrm{PR}\;\left(x^{*},t^{*}\;,x_{0}\right)$ *on* ${\left[0\;,\;t^{*}\right]}^{}$ *. Then, the following properties hold:*

***(i)***

(29) $$\left\|x\left(t^{*}\right)\right\|\in \left[\left|\left\|x^{*}\right\|-\left\|\int_{0}^{t}\Psi \left(t^{*},\;\tau \right)\xi \left(\tau \;,x\left(\tau \right)\right)d\tau \right\|\;\right|\;,\;\left\|x^{*}\right\|+\left\|\int_{0}^{t}\Psi \left(t^{*},\;\tau \right)\xi \left(x\left(\tau \right),u\left(\tau \right)\;,\;\tau \;\right)d\tau \right\|\right]$$

***(ii)***

(30) $$\left\|x\left(t^{*}\right)\right\|\leq \left\|x^{*}\right\|+{\left({\int_{0}^{t^{*}}\left\|\Psi \left(t^{*},\;\tau \right)\right\|}^{{}^{2}}d\tau \;\right)}^{1/2}{\left(\int_{0}^{t^{*}}{\left\|\xi \left(x\left(\tau \right),u\left(\tau \right)\;,\;\tau \;\right)\right\|}^{2}d\tau \right)}^{1/2}$$

***(iii)***

*If* $\left\|\Psi \left(t,\tau \right)\right\|\leq K_{\Psi }e^{-\rho_{\Psi }\left(t-\tau \right)}$ *with* $K_{\Psi }\;,\;\rho_{\Psi }\neq 0$ *then*

(31) $$\left\|x\left(t^{*}\right)\right\|\leq \left\|x^{*}\right\|+\frac{K_{\Psi }}{\sqrt{2\;\left|\rho_{\Psi }\right|}\;}\left|1-e^{-2\rho_{\Psi }t^{*}}\right|{}^{1/2}{\left(\int_{0}^{t^{*}}{\left\|\xi \left(x\left(\tau \right),u\left(\tau \right)\;,\;\tau \;\right)\right\|}^{2}d\tau \right)}^{1/2}$$

(32) $$\left\|x\left(t^{*}\right)\right\|\leq \left\|x^{*}\right\|+\frac{K_{\Psi }}{\;\left|\rho_{\Psi }\right|}\left|1-e^{-\rho_{\Psi }t^{*}}\right|\underset{0\leq \tau \leq t^{*}}{\sup}\left\|\xi \left(x\left(\tau \right),u\left(\tau \right)\;,\;\tau \;\right)\right\|$$

(33) $$\left\|x\left(t^{*}\right)\right\|\leq \left\|x^{*}\right\|+\underset{0\leq \tau \leq t^{*}}{\sup}\left\|\Psi \left(t^{*},\;\tau \right)\right\|\left(\int_{0}^{t^{*}}\left\|\xi \left(x\left(\tau \right),u\left(\tau \right)\;,\;\tau \;\right)\right\|d\tau \right)\leq \left\|x^{*}\right\|+K_{\Psi }\left|1-e^{-\rho_{\Psi }t^{*}}\right|\left(\int_{0}^{t^{*}}\left\|\xi \left(x\left(\tau \right),u\left(\tau \right)\;,\;\tau \;\right)\right\|d\tau \right)$$

*If, in addition,* $x^{*}\neq 0$ *then* $\underset{0\leq \tau \leq t^{*}}{\sup}\left\|\xi \left(x\left(\tau \right),u\left(\tau \right)\;,\;\tau \;\right)\right\|\leq \sigma_{\xi }x^{*}$ *and:*

(34) $$\left\|x\left(t^{*}\right)\right\|\leq \left(1+\frac{K_{\Psi }}{\left|\rho_{\Psi }\right|}\left|1-e^{-\rho_{\Psi }t^{*}}\right|\sigma_{\xi }\right)\;\left\|x^{*}\right\|$$

*If* $x^{*}=0$ *and* *x_0_ ≠ 0* *then* $\underset{0\leq \tau \leq t^{*}}{\sup}\left\|\xi \left(x\left(\tau \right),u\left(\tau \right)\;,\;\tau \;\right)\right\|\leq \sigma_{\xi 0}x_{0}$ *and:*

(35) $$\left\|x\left(t^{*}\right)\right\|\leq \frac{K_{\Psi }}{\left|\rho_{\Psi }\right|}\left|1-e^{-\rho_{\Psi }t^{*}}\right|\sigma_{\xi 0}\;\left\|x_{0}\right\|$$

**Proof.**  Since the current linearized system is $\mathrm{PR}\;\left(x^{*},t^{*}\;,x_{0}\right)$ then the controllability gramian of the current linearized system (1)–(2), subject to (3)–(4) on ${\left[0\;,\;t^{*}\right]}^{}$(that is, that associated with the pair (A(t) , b(t)) on ${\left[0\;,\;t^{*}\right]}^{}$) is:

(36) $$G_{c\left[0,t*\right]}\left(A\left(\tau \right)\;,b\left(\tau \right)\right)=\int_{0}^{t^{*}}\Psi \left(t^{*},\tau \right)b\left(\tau \right)b^{T}\left(\tau \right)\;\Psi^{T}\left(t^{*},\tau \right)d\tau$$

which is non-singular and the control law

(37) $$u\left(\tau \right)=b{}^{T}\left(\tau \right)\Psi^{T}\left(t,\tau \right)v\left(t^{*}\right)=b^{T}\left(\tau \right)\Psi^{T}\left(t^{*},\tau \right)G_{c\left[0,t*\right]}^{-1}\left(A\left(\tau \right)\;,b\left(\tau \right)\right)\left(x^{*}-\Psi \left(t^{*}\;,\;0\right)x_{0}\right);\;\tau \in \left[0\;,\;t^{*}\right]$$

transfers the initial state $x_{0}$ to $x\left(t^{*}\right)=x^{*}$ along the time interval ${\left[0\;,\;t^{*}\right]}^{}$ if ξ(τ , x(τ))≡0 in (1) for $\tau \in {\left[0\;,\;t^{*}\right]}^{{}^{}}$. If such a nonlinear contribution is non-zero then $x\left(t^{*}\right)=x^{*}+\int_{0}^{t}\Psi \left(t^{*},\;\tau \right)\xi \left(x\left(\tau \right),u\left(\tau \right)\;,\;\tau \;\right)d\tau$ so that (29) holds and Property (i) is proved. Property (ii) holds since (30) follows from H$\ddot{o}$lder´s inequality in the upper-bound of the right-hand-side of (29). Equations (31) and (33) follow from the right-hand side of (29) and (30) if Ψ(t,τ) is of exponential order but non-necessarily stable since $t^{*}$ is finite and Property (iii) is proved. On the other hand, Equations (34) and (35) follow directly from (32) under the given conditions. □

Sufficiency-type conditions which guarantee the non-singularity of the controllability gramian of the current linearized system in the case that that of the nominal one is non-singular is given in Appendix B.

## 3. Reachability and Approximate Reachability of the Current Time-Varying System under Unstructured Nonlinear Dynamics

**Theorem** **5.** *Define*$x\left(t^{*}\right)$*and*$x_{0}\left(t^{*}\right)$*as the current total state including parametrical disturbances and the nonlinear effects and the nominal linearized one, respectively, under the respective controls*u(t)*and*$u_{0}\left(t\right)$. *Then, the following properties hold:*

***(i)***

(38) $$x\left(t^{*}\right)-x_{0}\left(t^{*}\right)=\left(\Psi \left(t^{*},\;0\right)-e^{A_{0}t^{*}}\right)x_{0}\;+\int_{0}^{t*}\Psi \left(t^{*},\;\tau \right)\;\left(b\left(\tau \right)u\left(\tau \right)+\xi \left(\tau \;,\;x\left(\tau \right)\right)\right)d\tau -\int_{0}^{t*}e^{A_{0}\left(t^{*}-\tau \right)}\;b_{0}u_{0}\left(\tau \right)d\tau$$

***(ii)*** *Assume that the current linearized system (i.e., that resulting for* ξ(t , x(t))≡0 *)* *is* $\mathrm{PR}\;\left(x^{*},t^{*}\;,x_{0}\right)$ *and that* $u\left(t\right)=u^{*}\left(t\right)$ *;* $\forall t\in {\left[0\;,\;t^{*}\right]}^{}$ *achieves perfect state targeting* $x^{*}\left(t^{*}\right)=x^{*}$ *at* $t=t^{*}$ *. Then,*

(39) $$x^{*}-x_{0}\left(t^{*}\right)=\left(\Psi \left(t^{*},\;0\right)-e^{A_{0}t^{*}}\right)x_{0}+\int_{0}^{t*}\Psi \left(t^{*},\;\tau \right)\;b\left(\tau \right)u^{*}\left(\tau \right)d\tau -\int_{0}^{t*}e^{A_{0}\left(t^{*}-\tau \right)}\;b_{0}u_{0}\left(\tau \right)d\tau$$

***(iii)*** *Assume that the current linearized system (i.e., if* $\tilde{A}\left(t\right)=0$ *,* $\tilde{b}\left(t\right)=0$ *,* $\tilde{c}\left(t\right)=0$ *,* $\tilde{d}\left(t\right)=0$ *,* ξ(t, x(t))=0 ξ(t , x(t))≡0 *)* *is* $\mathrm{PR}\;\left(x^{*},t^{*}\;,x_{0}\right)$ *and that* $u_{0}\left(t\right)=u_{0}^{*}\left(t\right)$ *;* $\forall t\in {\left[0\;,\;t^{*}\right]}^{}$ *, Equation (12), achieves perfect state targeting* $x_{0}^{*}\left(t^{*}\right)=x^{*}$ *at* $t=t^{*}$ *. Then,*

(40) $$x\left(t^{*}\right)=\Psi \left(t^{*},\;0\right)x_{0}+\int_{0}^{t*}\Psi \left(t^{*},\;\tau \right)\;\left(b\left(\tau \right)u_{0}^{*}\left(\tau \right)+\xi \left(x\left(\tau \right),u\left(\tau \right)\;,\;\tau \;\right)\right)d\tau$$

**Proof.**  Note from (26) that, for initial conditions $x\left(0\right)=x_{0}$, $x\left(t^{*}\right)$ and $x_{0}\left(t^{*}\right)$ are given by:

(41) $$x\left(t^{*}\right)=\Psi \left(t^{*},\;0\right)x_{0}+\int_{0}^{t*}\Psi \left(t^{*},\;\tau \right)\;\left(b\left(\tau \right)u\left(\tau \right)+\xi \left(x\left(\tau \right),u\left(\tau \right)\;,\;\tau \;\right)\right)d\tau$$

(42) $$x_{0}\left(t^{*}\right)=e^{A_{0}t^{*}}x_{0}+\int_{0}^{t*}e^{A_{0}\left(t^{*}-\tau \right)}\;b_{0}u_{0}\left(\tau \right)d\tau$$

leading directly to (38). Property (i) is proved. On the other hand, if the current linearized is $\mathrm{PR}\;\left(x^{*},t^{*}\;,x_{0}\right)$ and that $u\left(t\right)=u^{*}\left(t\right)$; $\forall t\in {\left[0\;,\;t^{*}\right]}^{}$ achieves perfect state targeting $x^{*}\left(t^{*}\right)=x^{*}$ at $t=t^{*}$. Then:

(43) $$x\left(t^{*}\right)-x^{*}=\int_{0}^{t*}\Psi \left(t^{*},\;\tau \right)\;\xi \left(x\left(\tau \right),u\left(\tau \right)\;,\;\tau \;\right)d\tau$$

and replacing it into (38) and simplifying the resulting equation yields (39). Property (iii) follows directly from the fact that $u_{0}\left(t\right)=u_{0}^{*}\left(t\right)$; $\forall t\in {\left[0\;,\;t^{*}\right]}^{}$ achieves nominal linearized perfect state targeting $x_{0}^{*}\left(t^{*}\right)=x^{*}$ at $t=t^{*}$ assumed it is $\mathrm{PR}\;\left(x^{*},t^{*}\;,x_{0}\right)$ and then:

(44) $$x^{*}=e^{A_{0}t^{*}}x_{0}+\int_{0}^{t*}e^{A_{0}\left(t^{*}-\tau \right)}\;b_{0}u_{0}^{*}\left(\tau \right)d\tau$$

replaced in (38) gives (40). □

The following result provides a worst-case estimate of the Euclidean norm of the sate norm versus time of the current system on the reachability interval ${\left[0\;,\;t^{*}\right]}^{}$ of the current linearized system, which is not assumed to be necessarily stable, if the control law (37) is used and the targeting objective at time $t^{*}$ is scheduled for the current linearized system which is point reachable and whose complete time-varying parameterization is exactly known for all time.

**Theorem** **6.** *Assume that:*

*A1)*  *The current linearized system is* $\mathrm{PR}\;\left(x^{*},t^{*}\;,x_{0}\right)$ *and that the* *perfect state targeting objective* $x^{*}\left(t^{*}\right)=x^{*}$ *is scheduled for it.*
*A2)*  Ψ(t,τ)  *is of exponential order (although the current linearized system is not assumed necessarily stable) and its Euclidean norm satisfies*$K_{\Psi 0}e^{-\rho_{\Psi 0}\left(t-\tau \right)}\leq {\left\|\Psi \left(t,\tau \right)\right\|}_{2}\leq K_{\Psi }e^{-\rho_{\Psi }\left(t-\tau \right)}$*;*$\forall t\left(\geq \tau \right)\;,\;\tau \in R_{0+}$*for some real constants*$\rho_{\Psi 0}\geq \frac{\rho_{\Psi }}{2}$*,*$\rho_{\Psi }$*,*$K_{\Psi 0}>0$*and*$K_{\Psi }\geq 1$.
*A3)*  $\max\;\left(\left|1-e^{-\rho_{\Psi }t^{*}}\right|\;,\;\left|e^{\left|\rho_{\Psi }\right|t^{*}}-1\right|\right)<\frac{\left|\rho_{\Psi }\right|}{K_{\Psi }}$
*A4)*  *The point reachability control law (37) is injected to the current system.*
*A5)*  *The nonlinear contribution to the dynamics related to the nominal current linearized system satisfies the worst-case growing condition:*
  (45) $$\left\|\xi \left(x\left(t\right)\;,u\left(t\right)\right)\right\|\leq K_{\xi x}\underset{0\leq \tau \leq t}{\sup}{\left\|x\left(\tau \right)\right\|}_{2}^{2}+K_{\xi u}\underset{0\leq \tau \leq t}{\sup}{\left\|u\left(\tau \right)\right\|}_{2}^{2};\tau \in {\left[0\;,\;t\right]}^{};t\in {\left[0\;,\;t^{*}\right]}^{}$$
  *for some real constants*$K_{\xi x}\geq 0$*and*$K_{\xi u}\geq 0$.
*A6)*  $K_{\xi x}<\frac{\left|\rho_{\Psi }\right|}{\alpha \;K_{\Psi }{\bar{M}}_{x}}$
  *where:*
  (46) $${\bar{M}}_{x}=\frac{K_{\Psi }\left|\rho_{\Psi }\right|}{\left|\rho_{\Psi }\right|-\alpha K_{\Psi }\theta_{\xi }}(e^{-\rho_{\Psi }t^{*}}{\left\|x_{0}\right\|}_{2}+\frac{K_{\Psi }\alpha \gamma }{\left|\rho_{\Psi }\right|\beta }\underset{0\leq t\leq t^{*}}{\sup}\left\|b\left(t\right)\right\|{}_{2}^{2}\;\left(\frac{\;\left({\left\|\;x^{*}\right\|}_{2}+K_{\Psi }e^{-\rho_{\Psi }t^{*}}{\left\|x_{0}\right\|}_{2}\right)}{\lambda_{\min}\;{\left(\int_{0}^{t^{*}}b\left(t\right)\;b^{T}\left(t\right)\;dt\right)}^{}}\right)\;+\frac{\alpha \gamma^{2}\;K_{\xi u}K_{\Psi }^{2}}{\left|\rho_{\Psi }\right|\beta^{2}}\;\left(\frac{{\left\|b\left(t\right)\right\|}_{2}^{2}\;{\left({\left\|\;x^{*}\right\|}_{2}+K_{\Psi }e^{-\rho_{\Psi }t^{*}}{\left\|x_{0}\right\|}_{2}\right)}^{2}}{\lambda_{\min}^{2}\;{\left(\int_{0}^{t^{*}}b\left(t\right)\;b^{T}\left(t\right)dt\right)}^{}}\right)\;)$$
  *with*$\theta_{\xi }=\inf\;\left(\theta \in R_{0+}:K_{\xi x}\leq \frac{\theta }{\underset{0\leq t\leq t^{*}}{\sup}{\left\|x\left(t\right)\right\|}_{2}}\right)$*,*$\alpha =\alpha \left(\rho_{\Psi }\;,t^{*}\right)=\max\;\left(\left|1-e^{-\rho_{\Psi }t^{*}}\right|\;,\;\left|e^{\left|\rho_{\Psi }\right|t^{*}}-1\right|\right)$*,*$\gamma =\gamma \left(t^{*}\right)=\max\;\left(1\;,\;e^{-\rho_{\Psi }t^{*}}\right)$*, and*$\beta \left(\rho_{\Psi }\;,t^{*}\right)=\{\;\begin{array}{c} K_{\Psi 0}^{2}\;\;\;if\;\rho_{\Psi 0}\leq 0\\ K_{\Psi 0}^{2}e^{-2\rho_{\Psi 0}t^{*}}\;\mathrm{if}\;\rho_{\Psi 0}>0 \end{array}$.

*Then,*$\underset{0\leq t\leq t^{*}}{\sup}{\left\|x\left(t\right)\right\|}_{2}\leq {\bar{M}}_{x}$.

**Proof.**  Note from (31) that since the controllability grammian and its inverse are symmetric on any time interval, denoting with $\lambda_{\min}\left(.\right)$ and $\lambda_{\max}\left(.\right)$ the minimum and maximum eigenvalues of the square symmetric-(.) matrix, one has:

(47) $${\left\|u\left(t\right)\right\|}_{2}\leq {\left\|b\left(t\right)\right\|}_{2}\;{\left\|\Psi \left(t^{*},t\right)\right\|}_{2}\;{\left\|G_{c\left[0,t*\right]}^{-1}\left(A\left(t\right)\;,b\left(t\right)\right)\right\|}_{2}{\left\|\;x^{*}-\Psi \left(t^{*}\;,\;0\right)x_{0}\right\|}_{2}\;=\frac{{\left\|b\left(t\right)\right\|}_{2}\;\underset{0\leq t\leq t^{*}}{\sup}{\left\|\Psi \left(t^{*},t\right)\right\|}_{2}\;{\left\|\;x^{*}-\Psi \left(t^{*}\;,\;0\right)x_{0}\right\|}_{2}}{\lambda_{\min}\;{\left(G_{c\left[0,t*\right]}\left(A\left(t\right)\;,b\left(t\right)\right)\right)}^{}}\;\leq \frac{{\left\|b\left(t\right)\right\|}_{2}\;\underset{0\leq t\leq t^{*}}{\sup}\;\lambda_{\max}^{1/2}\;\left(\Psi \left(t^{*},t\right)\;\Psi^{T}\left(t^{*},t\right)\right)\;{\left\|\;x^{*}-\Psi \left(t^{*}\;,\;0\right)x_{0}\right\|}_{2}}{\underset{0\leq t\leq t^{*}}{\inf}\lambda_{\min}\;{\left(\Psi \left(t^{*},t\right)\;\Psi^{T}\left(t^{*},t\right)\right)}^{}\lambda_{\min}\;{\left(\int_{0}^{t^{*}}b\left(t\right)\;b^{T}\left(t\right)dt\right)}^{}}\;;\;\forall t\in {\left[0\;,\;t^{*}\right]}^{}$$

Since $\lambda_{\max}^{1/2}\;\left(\Psi \left(t,\tau \right)\;\Psi^{T}\left(t,\tau \right)\right)={\left\|\Psi \left(t,\tau \right)\right\|}_{2}\leq K_{\Psi }e^{-\rho_{\Psi }\left(t-\tau \right)}$ and $\lambda_{\min}\;\left(\Psi \left(t,\tau \right)\;\;\Psi^{T}\left(t,\tau \right)\right)\geq K_{\Psi 0}^{2}e^{-2\rho_{{}_{\Psi 0}}\left(t-\tau \right)}$; $\forall t\left(\geq \tau \right)\;,\;\tau \in R_{0+}$ and then:

(48) $$\beta =\beta \left(\rho_{\Psi 0}\;,t^{*}\right)=\underset{0\leq t\leq t^{*}}{\inf}\lambda_{\min}\;{\left(\Psi \left(t^{*},t\right)\;\Psi^{T}\left(t^{*},t\right)\right)}^{}=\{\;\begin{array}{c} K_{\Psi 0}^{2}\;\;\;if\;\rho_{\Psi 0}\leq 0\\ K_{\Psi 0}^{2}e^{-2\rho_{\Psi 0}t^{*}}\;\mathrm{if}\;\rho_{\Psi 0}>0 \end{array}$$

Note that, since $\rho_{\Psi 0}\geq \frac{\rho_{\Psi }}{2}$, then if $\rho_{\Psi 0}\leq 0\Rightarrow \rho_{\Psi }\leq 0$ so that the current linearized system is either unstable or critically stable and $\rho_{\Psi }>0\Rightarrow \rho_{\Psi 0}>0$ so that the system is exponentially stable. Define also $\gamma =\gamma \left(t^{*}\right)=\max\;\left(1\;,\;e^{-\rho_{\Psi }t^{*}}\right)$. Then, one gets from (48) into (47) that:

(49) $${\left\|u\left(t\right)\right\|}_{2}\leq \frac{K_{\Psi }\gamma }{\beta \;}\frac{{\left\|b\left(t\right)\right\|}_{2}\;{\left\|\;x^{*}-\Psi \left(t^{*}\;,\;0\right)x_{0}\right\|}_{2}}{\lambda_{\min}\;{\left(\int_{0}^{t^{*}}b\left(t\right)\;b^{T}\left(t\right)dt\right)}^{}}\leq \frac{K_{\Psi }\gamma }{\beta \;}(\frac{{\left\|b\left(t\right)\right\|}_{2}\;\left({\left\|\;x^{*}\right\|}_{2}+K_{\Psi }e^{-\rho_{\Psi }t^{*}}{\left\|x_{0}\right\|}_{2}\right)}{\lambda_{\min}\;{\left(\int_{0}^{t^{*}}b\left(t\right)\;b^{T}\left(t\right)dt\right)}^{}});\;\;\forall t\in {\left[0\;,\;t^{*}\right]}^{}$$

Define $M_{x}=M_{x}\left(t^{*}\right)=\underset{0\leq t\leq t^{*}}{\sup}{\left\|x\left(t\right)\right\|}_{2}\leq \theta_{\xi }/K_{\xi x}$ for some $\theta_{\xi }\in R_{0+}$. Thus, one has from (45) into (41) that:

(50) $$M_{x}=\underset{0\leq t\leq t^{*}}{\sup}{\left\|x\left(t\right)\right\|}_{2}\leq \underset{0\leq t\leq t^{*}}{\sup}{\left\|\Psi \left(t,\;0\right)\right\|}_{2}{\left\|x_{0}\right\|}_{2}+\underset{0\leq t\leq t^{*}}{\sup}\underset{0\leq \tau \leq t}{\sup}{\left\|\int_{0}^{t}\Psi \left(t,\;\tau \right)\;d\tau \right\|}_{2}\;\times (K_{\xi x}M_{x}^{2}+\underset{0\leq t\leq t^{*}}{\sup}\left({\left\|b\left(t\right)\right\|}_{2}+K_{\xi u}\underset{0\leq t\leq t^{*}}{\sup}{\left\|u\left(t\right)\right\|}_{2}\right)\;\underset{0\leq t\leq t^{*}}{\sup}{\left\|u\left(t\right)\right\|}_{2})\;\leq K_{\Psi }\max\;\left(\left|1-e^{-\rho_{\Psi }t^{*}}\right|\;,\;\left|e^{\left|\rho_{\Psi }\right|t^{*}}-1\right|\right)\left[\frac{1}{\left|\rho_{\Psi }\right|}(K_{\xi x}\theta_{\xi }M_{x}^{2}+\underset{0\leq t\leq t^{*}}{\sup}\left({\left\|b\left(t\right)\right\|}_{2}+K_{\xi u}\underset{0\leq t\leq t^{*}}{\sup}{\left\|u\left(t\right)\right\|}_{2}\right)\;\underset{0\leq t\leq t^{*}}{\sup}{\left\|u\left(t\right)\right\|}_{2})\right]\;+K_{\Psi }e^{-\rho_{\Psi }t^{*}}{\left\|x_{0}\right\|}_{2}$$

so that, since $K_{\xi x}M_{x}^{2}\leq M_{x}$, one gets by defining $\alpha =\alpha \left(\rho_{\Psi }\;,t^{*}\right)=\max\;\left(\left|1-e^{-\rho_{\Psi }t^{*}}\right|\;,\;\left|e^{\left|\rho_{\Psi }\right|t^{*}}-1\right|\right)$ and after re-arranging factors of $M_{x}$ and re-allocating them to the left-hand-side of the above equation:

(51) $$M_{x}\leq {\left(1-\frac{\alpha \;K_{\Psi }}{\left|\rho_{\Psi }\right|}\right)}^{-1}(K_{\Psi }e^{-\rho_{\Psi }t^{*}}{\left\|x_{0}\right\|}_{2}\;+\frac{K_{\Psi }\alpha }{\left|\rho_{\Psi }\right|}\;\left[\;\underset{0\leq t\leq t^{*}}{\sup}\left({\left\|b\left(t\right)\right\|}_{2}+K_{\xi u}\underset{0\leq t\leq t^{*}}{\sup}{\left\|u\left(t\right)\right\|}_{2}\right)\;\underset{0\leq t\leq t^{*}}{\sup}{\left\|u\left(t\right)\right\|}_{2}\right]\;)$$

The substitution of (49) into (51) yields $\underset{0\leq t\leq t^{*}}{\sup}{\left\|x\left(t\right)\right\|}_{2}\leq M_{x}\leq {\bar{M}}_{x}$ with ${\bar{M}}_{x}$ defined in (46) provided that $\left|\rho_{\Psi }\right|>\alpha K_{\Psi }\theta_{\xi }=K_{\Psi }\theta_{\xi }\max\;\left(\left|1-e^{-\rho_{\Psi }t^{*}}\right|\;,\;\left|e^{\left|\rho_{\Psi }\right|t^{*}}-1\right|\right)$. Note that the condition $K_{\xi x}M_{x}\leq \theta_{\xi }<\frac{\left|\rho_{\Psi }\right|}{\alpha \;K_{\Psi }}$ always hold for some $\theta_{\xi }\geq 0$ if $K_{\xi x}$ is small enough to satisfy $K_{\xi x}<\frac{\left|\rho_{\Psi }\right|}{\alpha \;K_{\Psi }\underset{0\leq t\leq t^{*}}{\sup}{\left\|x\left(t\right)\right\|}_{2}}$. Such a constraint is guaranteed by looking for a lower bound than $\frac{\left|\rho_{\Psi }\right|}{\alpha \;K_{\Psi }\underset{0\leq t\leq t^{*}}{\sup}{\left\|x\left(t\right)\right\|}_{2}}$ by using $\underset{0\leq t\leq t^{*}}{\sup}{\left\|x\left(t\right)\right\|}_{2}\leq M_{x}$ via (51) resulting to be $K_{\xi x}<\frac{\left|\rho_{\Psi }\right|}{\alpha \;K_{\Psi }{\bar{M}}_{x}}$. □

The results of Theorem 6 are now specialized for the case when the targeting objective is an asymptotic objective, that is, scheduled for arbitrarily large time $t^{*}$.

**Corollary** **1.** *Assume that the assumptions of Theorem 6 hold with* $\rho_{\Psi }>0$ *(implying that the current linearized system is exponentially stable) so that the Assumption A3 becomes:*

$$1-e^{-\rho_{\Psi }t^{*}}<\frac{\left|\rho_{\Psi }\right|}{K_{\Psi }}$$

*and that:*

$$\underset{t^{*}\to \infty }{\lim\;\inf}\left[\beta \left(\rho_{\Psi }\;,t^{*}\right)\lambda_{\min}\int_{0}^{t^{*}}b\left(t\right)b^{T}\left(t\right)dt\right]\geq \beta_{1}>0$$

$$\underset{t^{*}\to \infty }{\lim\;\sup}\left[\beta \left(\rho_{\Psi }\;,t^{*}\right)\lambda_{\min}\int_{0}^{t^{*}}b\left(t\right)b^{T}\left(t\right)dt\right]\leq \beta_{2}<+\infty$$

*Then:*

(52) $$\underset{t^{*}\to \infty }{\lim\;\sup}\left({\bar{M}}_{x}\left(t^{*}\right)\;\;-\frac{K_{\Psi }^{2}\rho_{\Psi }}{{\left(\rho_{\Psi }-K_{\Psi }\theta_{\xi }\right)}^{}\rho_{\Psi }\beta_{1}}\underset{0\leq t\leq t^{*}}{\sup}\left\|b\left(t\right)\right\|{}_{2}^{2}\left(\;1+\frac{2K_{\Psi }K_{\xi u}{\left\|x^{*}\right\|}_{2}}{\beta_{1}}\;\right)\;{\left\|x^{*}\right\|}_{2}\right)\leq 0$$

(53) $$\underset{K_{\xi \;u\to 0}}{\lim}\left[\underset{t^{*}\to \infty }{\lim\;\sup}\left({\bar{M}}_{x}\left(t^{*}\right)\;\;-\frac{K_{\Psi }^{2}\rho_{\Psi }}{{\left(\rho_{\Psi }-K_{\Psi }\theta_{\xi }\right)}^{}\rho_{\Psi }\beta_{1}}\underset{0\leq t\leq t^{*}}{\sup}\left\|b\left(t\right)\right\|{}_{2}^{2}\;{\left\|x^{*}\right\|}_{2}\right)\right]\;\leq 0$$

**Proof.**  First, note that (46) can be rewritten equivalently as follows after grouping terms:

(54) $${\bar{M}}_{x}={\bar{M}}_{x}\left(t^{*}\right)\;=\frac{K_{\Psi }\rho_{\Psi }}{\rho_{\Psi }-\alpha K_{\Psi }\theta_{\xi }}[\left(1+\frac{\alpha \gamma K_{\Psi }^{2}}{\rho_{\Psi }\beta \lambda_{\min}\;{\left(\int_{0}^{t^{*}}b\left(t\right)\;b^{T}\left(t\right)dt\right)}^{}}\left(\;1+\frac{2K_{\Psi }^{2}\gamma \;K_{\xi u}e^{-\rho_{\Psi }t^{*}}{\left\|x_{0}\right\|}_{2}}{\beta \lambda_{\min}\;{\left(\int_{0}^{t^{*}}b\left(t\right)\;b^{T}\left(t\right)dt\right)}^{}}\;\right)\frac{\underset{0\leq t\leq t^{*}}{\sup}\;\left\|b\left(t\right)\right\|{}_{2}^{2}\;}{\lambda_{\min}\;{\left(\int_{0}^{t^{*}}b\left(t\right)\;b^{T}\left(t\right)dt\right)}^{}}\;\right)\;e^{-\rho_{\Psi }t^{*}}{\left\|x_{0}\right\|}_{2}\;+\;\left(\frac{K_{\Psi }\alpha \gamma }{\rho_{\Psi }\beta \lambda_{\min}\;{\left(\int_{0}^{t^{*}}b\left(t\right)\;b^{T}\left(t\right)dt\right)}^{}}\underset{0\leq t\leq t^{*}}{\sup}\left\|b\left(t\right)\right\|{}_{2}^{2}\left(\;1+\frac{2\gamma K_{\Psi }K_{\xi u}{\left\|x^{*}\right\|}_{2}}{\beta \lambda_{\min}\;{\left(\int_{0}^{t^{*}}b\left(t\right)\;b^{T}\left(t\right)dt\right)}^{}}\;\right)\;\right)\;{\left\|x^{*}\right\|}_{2}]$$

Under the given assumptions, one has from (54) that, since $\rho_{\Psi }>0$, it follows that $\alpha \left(\rho_{\Psi }\;,t^{*}\right)=1-e^{-\rho_{\Psi }t^{*}}\to 0$ as $t^{*}\to \infty$ and γ=1, and then (52)–(53) hold. □

Another elementary targeting error estimate result follows below for the case when the current linearized system arises perfect targeting and the nonlinear disturbance grows slower than some power of the state norm.

**Theorem** **7.** *Let Assumptions A1 to A4 of Theorem 6 to hold and the constraint of A5 is replaced with the following one for some real constant*μ∈(1 , 2):

(55) $$\left\|\xi \left(x\left(t\right)\;\right)\right\|\leq K_{\xi x}{\left\|x\left(t\right)\right\|}_{2}^{\mu };t\in {\left[0\;,\;t^{*}\right]}^{}$$

*Assume also that* $K_{\xi x}$ *is small enough such that the condition C1 below holds:*

*C1)*

(56) $$K_{\xi x}\leq \underset{0\leq t\leq t^{*}}{\inf}\frac{1}{K_{\Psi }{\left|1-e^{-\left(\mu -1\right)\rho_{\Psi }t^{*}/\mu }\right|}^{{}^{\mu }}}{\left(\frac{\left(\mu -1\right){\left|\rho_{\Psi }\right|}^{\mu /\left(\mu -1\right)}}{\mu }\right)}^{\mu }\frac{\left\|\xi \left(x\left(t\right)\;\right)\right\|{}^{1/\mu }}{{\left(\int_{0}^{t^{*}}\left\|\xi \left(x\left(\sigma \right)\right)\right\|{}_{{}_{2}}^{{}^{1/\mu }}d\sigma \right)}^{\mu }}$$

*Then, if the targeting control law for the current linearized system (37) is applied to the current system, one has the following targeting error estimate:*

(57) $$\left\|x\left(t^{*}\right)-x^{*}\right\|{}_{{}_{2}}^{{}^{q}}\leq 4\frac{K_{\Psi }^{2}}{{\left|\rho_{\Psi }\right|}^{2}}\;{\left(\frac{qK_{\Psi }K_{\xi x}}{\left(q-1\right)\;{\left|\rho_{\Psi }\right|}^{q/\left(q-1\right)}}\right)}^{q}{\left|1-e^{-\left(q-1\right)\rho_{\Psi }t^{*}/q}\right|}^{{}^{q}}{\left|1-e^{-2\rho_{\Psi }t^{*}}\right|}^{2}\;\times \left({\left\|x_{0}\right\|}_{2}^{2}+t^{*2}\frac{K_{\Psi }^{3}\gamma }{{\left|\rho_{\Psi }\right|}^{2}\beta }\underset{0\leq t\leq t^{}}{\sup}{\left\|b\left(\sigma \right)\right\|}_{2}^{3}\;\frac{{\left({\left\|\;x^{*}\right\|}_{2}+K_{\Psi }e^{-\rho_{\Psi }t^{*}}{\left\|x_{0}\right\|}_{2}\right)}^{2}}{\lambda {}_{\min}^{2}\;{\left(\int_{0}^{t^{*}}b\left(t\right)\;b^{T}\left(t\right)dt\right)}^{}}\right)\;+2\;{\left(\frac{qK_{\Psi }K_{\xi x}}{\;\left(q-1\right){\left|\rho_{\Psi }\right|}^{q/\left(q-1\right)}}\right)}^{q}{\left|1-e^{-\left(q-1\right)\rho_{\Psi }t^{*}/q}\right|}^{{}^{q}}\frac{\left\|\xi \left(x\left(t\right)\;\right)\right\|{}^{2/\mu }}{K_{\xi x}^{2}}$$

**Proof.**  Note from (55) and condition C1 that;

(58) $$0\leq \left\|\xi \left(x\left(t\right)\;\right)\right\|{}^{1/\mu }-K_{\xi x}K_{\Psi }{\left(\frac{\mu }{\;\left(\mu -1\right){\left|\rho_{\Psi }\right|}^{\mu /\left(\mu -1\right)}}\right)}^{\mu }{\left|1-e^{-\left(\mu -1\right)\rho_{\Psi }t^{*}/\mu }\right|}^{{}^{\mu }}\;{\left(\int_{0}^{t}\left\|\xi \left(x\left(\sigma \right)\right)\right\|{}_{{}_{2}}^{{}^{1/\mu }}d\sigma \right)}^{\mu }\;\leq \left\|\xi \left(x\left(t\right)\;\right)\right\|{}^{1/\mu }-K_{\xi x}{\left(\int_{0}^{t}\left\|\int_{0}^{\tau }\Psi \left(\tau ,\sigma \right)d\sigma \right\|{}_{2}^{\left(\mu -1\right)/\mu }d\tau \right)}^{\mu /\left(\mu -1\right)}\;{\left(\int_{0}^{t}\left\|\xi \left(x\left(\sigma \right)\right)\right\|{}_{{}_{2}}^{{}^{1/\mu }}d\sigma \right)}^{\mu }\;\leq \left\|\xi \left(x\left(t\right)\;\right)\right\|{}^{1/\mu }-K_{\xi x}{\left\|\int_{0}^{t}\int_{0}^{\tau }\Psi \left(\tau ,\sigma \right)\xi \left(x\left(\sigma \right)\right)d\sigma d\tau \right\|}_{2}\leq K_{\xi x}\|\int_{0}^{t}\Psi \left(\tau ,0\right)x_{0}+\int_{0}^{\tau }\left(\Psi \left(\tau ,\sigma \right)b\left(\sigma \right)\;u\left(\sigma \right)\right)d\sigma d\tau \|;\;\;t\in {\left[0\;,\;t^{*}\right]}^{}$$

so that:

(59) $${\left\|\int_{0}^{t}\int_{0}^{\tau }\Psi \left(\tau ,\sigma \right)\xi \left(x\left(\sigma \right)\right)d\sigma d\tau \right\|}_{2}\leq \frac{\left\|\xi \left(x\left(t\right)\;\right)\right\|{}^{1/\mu }}{K_{\xi x}};\;t\in {\left[0\;,\;t^{*}\right]}^{}$$

On the other hand. since the current linearized system is $\mathrm{PR}\;\left(x^{*},t^{*}\;,x_{0}\right)$ and its perfect state targeting objective $x^{*}\left(t^{*}\right)=x^{*}$ at time $t=t^{*}$ is achieved by the control law (37) it turns out, by using (55) and H$\ddot{o}$lder´s inequality in (56) with q=2/μ>1 and $p=q/\left(q-1\right)=\frac{2}{2-\mu }$, that the current system satisfies:

(60) $${\left\|x\left(t^{*}\right)-x^{*}\right\|}_{2}={\left\|\;\int_{0}^{t*}\Psi \left(t^{*}\;,\tau \right)\;\xi \left(x\left(\tau \right)\right)\;d\tau \right\|}_{2}\leq {\left(\int_{0}^{t*}\left\|\Psi \left(t^{*}\;,\tau \right)\;d\tau \right\|{}_{2}^{{}^{\left(q-1\right)/q}}\right)}^{{}^{q/\left(q-1\right)}}\left(\int_{0}^{t*}\;\left\|\xi \left(x\left(\tau \right)\right)\;d\tau \right\|{}_{2}^{{}^{q}}\right){}^{1/q}\;\leq \frac{K_{\Psi }K_{\xi x}}{{\left|\rho_{\Psi }\right|}^{q/\left(q-1\right)}\left(q-1\right)}\left|1-e^{-\left(q-1\right)\rho_{\Psi }t^{*}/q}\right|{}^{q/\left(q-1\right)}{\left(\int_{0}^{t^{*}}{\left\|\;x\left(\tau \right)\right\|}_{2}^{\mu q}d\tau \right)}^{1/q}$$

Thus:

(61) $$\left\|x\left(t^{*}\right)-x^{*}\right\|{}_{{}_{2}}^{{}^{q}}\leq {\left(\frac{qK_{\Psi }K_{\xi x}}{\;\left(q-1\right){\left|\rho_{\Psi }\right|}^{q/\left(q-1\right)}}\right)}^{q}{\left|1-e^{-\left(q-1\right)\rho_{\Psi }t^{*}/q}\right|}^{{}^{q}}\left(\int_{0}^{t^{*}}{\left\|x\left(\tau \right)\right\|}_{2}^{2}d\tau \right)\;\leq {\left(\frac{qK_{\Psi }K_{\xi x}}{\;\left(q-1\right){\left|\rho_{\Psi }\right|}^{q/\left(q-1\right)}}\right)}^{q}{\left|1-e^{-\left(q-1\right)\rho_{\Psi }t^{*}/q}\right|}^{{}^{q}}\left(\int_{0}^{t^{*}}{\left\|x\left(\tau \right)\right\|}_{2}^{2}d\tau \right)$$

(62) $$\begin{array}{l} \leq {\left(\frac{qK_{\Psi }K_{\xi x}}{\left(q-1\right)\;{\left|\rho_{\Psi }\right|}^{q/\left(q-1\right)}}\right)}^{q}{\left|1-e^{-\left(q-1\right)\rho_{\Psi }t^{*}/q}\right|}^{{}^{q}}\\ \;\;\;\;\;\;\times \left(\left(\int_{0}^{t^{*}}{\left\|\Psi \left(\tau ,0\right)x_{0}+\int_{0}^{\tau }\left(\Psi \left(\tau ,\sigma \right)b\left(\sigma \right)\;u\left(\sigma \right)+\int_{0}^{\tau }\Psi \left(\tau ,\sigma \right)\xi \left(x\left(\sigma \right)\right)\right)d\sigma \right\|}_{2}^{2}\right)d\tau \right) \end{array}$$

and by using (59) for the nonlinear dynamics contribution upper-bound and (49) for the control upper-bound, one gets:

(63) $$\left\|x\left(t^{*}\right)-x^{*}\right\|{}_{{}_{2}}^{{}^{q}}-2\;{\left(\frac{qK_{\Psi }K_{\xi x}}{\;\left(q-1\right){\left|\rho_{\Psi }\right|}^{q/\left(q-1\right)}}\right)}^{q}{\left|1-e^{-\left(q-1\right)\rho_{\Psi }t^{*}/q}\right|}^{{}^{q}}\frac{\left\|\xi \left(x\left(t\right)\;\right)\right\|{}^{2/\mu }}{K_{\xi x}^{2}}\;\leq \left\|x\left(t^{*}\right)-x^{*}\right\|{}_{{}_{2}}^{{}^{q}}-2\;{\left(\frac{qK_{\Psi }K_{\xi x}}{\;\left(q-1\right){\left|\rho_{\Psi }\right|}^{q/\left(q-1\right)}}\right)}^{q}{\left|1-e^{-\left(q-1\right)\rho_{\Psi }t^{*}/q}\right|}^{{}^{q}}{\left(\int_{0}^{t^{*}}\int_{0}^{\tau }\Psi \left(\tau ,\sigma \right)\xi \left(x\left(\sigma \right)\right)d\sigma d\tau \right)}^{2}\;\leq 2\;{\left(\frac{qK_{\Psi }K_{\xi x}}{\left(q-1\right)\;{\left|\rho_{\Psi }\right|}^{q/\left(q-1\right)}}\right)}^{q}{\left|1-e^{-\left(q-1\right)\rho_{\Psi }t^{*}/q}\right|}^{{}^{q}}\left(\left(\int_{0}^{t^{*}}{\left\|\Psi \left(\tau ,0\right)x_{0}+\int_{0}^{\tau }\left(\Psi \left(\tau ,\sigma \right)b\left(\sigma \right)\;u\left(\sigma \right)\right)d\sigma \right\|}_{2}^{2}\right)d\tau \right)\;\leq 4\;{\left(\frac{qK_{\Psi }K_{\xi x}}{\left(q-1\right)\;{\left|\rho_{\Psi }\right|}^{q/\left(q-1\right)}}\right)}^{q}{\left|1-e^{-\left(q-1\right)\rho_{\Psi }t^{*}/q}\right|}^{{}^{q}}\left(\left(\int_{0}^{t^{*}}{\left\|\Psi \left(\tau ,0\right)\right\|}_{2}d\tau \right){}^{2}{\left\|x_{0}\right\|}_{2}^{2}+\left(\int_{0}^{t^{*}}\int_{0}^{\tau }\Psi \left(\tau ,\sigma \right)b\left(\sigma \right)\;u\left(\sigma \right)d\sigma d\tau \right){}^{2}\right)\;\leq 4\frac{K_{\Psi }^{2}}{{\left|\rho_{\Psi }\right|}^{2}}\;{\left(\frac{qK_{\Psi }K_{\xi x}}{\left(q-1\right)\;{\left|\rho_{\Psi }\right|}^{q/\left(q-1\right)}}\right)}^{q}{\left|1-e^{-\left(q-1\right)\rho_{\Psi }t^{*}/q}\right|}^{{}^{q}}{\left|1-e^{-2\rho_{\Psi }t^{*}}\right|}^{2}\times \left({\left\|x_{0}\right\|}_{2}^{2}+t^{*2}\frac{K_{\Psi }^{2}}{{\left|\rho_{\Psi }\right|}^{2}}\underset{0\leq \sigma \leq t^{}}{\sup}{\left\|b\left(\sigma \right)\right\|}_{2}^{2}\;{\left\|u\left(\sigma \right)\right\|}_{2}^{2}\right)\;\leq 4\frac{K_{\Psi }^{2}}{{\left|\rho_{\Psi }\right|}^{2}}\;{\left(\frac{qK_{\Psi }K_{\xi x}}{\left(q-1\right)\;{\left|\rho_{\Psi }\right|}^{q/\left(q-1\right)}}\right)}^{q}{\left|1-e^{-\left(q-1\right)\rho_{\Psi }t^{*}/q}\right|}^{{}^{q}}{\left|1-e^{-2\rho_{\Psi }t^{*}}\right|}^{2}\;\times \left({\left\|x_{0}\right\|}_{2}^{2}+t^{*2}\frac{K_{\Psi }^{3}\gamma }{{\left|\rho_{\Psi }\right|}^{2}\beta }\underset{0\leq \sigma \leq t^{}}{\sup}{\left\|b\left(\sigma \right)\right\|}_{2}^{3}\;\frac{{\left({\left\|\;x^{*}\right\|}_{2}+K_{\Psi }e^{-\rho_{\Psi }t^{*}}{\left\|x_{0}\right\|}_{2}\right)}^{2}}{\lambda {}_{\min}^{2}\;{\left(\int_{0}^{t^{*}}b\left(t\right)\;b^{T}\left(t\right)dt\right)}^{}}\right)$$

and the result follows directly from (63). □

## 4. Output Reachability, Output Approximate Reachability and Practical Constraints

### 4.1. Output Reachability and Output Approximate Reachability

It is direct to extend Definitions 1–4 to the various parallel concepts from (state) reachability of output point reachability and approximate output reachability $\mathrm{OPR}\;\left(y^{*},t^{*}\;,x_{0}\right)$ and $\mathrm{AOPR}\;\left(y^{*},t^{*}\;,x_{0}\right)$ in a direct way when the targeted objective at time $t^{*}$ is just to prefix an output value $y\left(t^{*}\right)=y^{*}=c^{T}\left(t^{*}\right)x^{*}$ to be either exactly or approximately targeted. The basic ideas are easy to extend from the former section so that we only give some guidelines. Suppose for the shake of simplicity that the direct input-output interconnection gain $d\left(t\right)=d_{0}\equiv 0$. Basically, the output controllability gramians on ${\left[0\;,\;t^{*}\right]}^{}$ of the current and nominal linearized systems are, respectively:

$G_{oc\left[0,t*\right]}\left(c^{T}\left(t\right),A\left(t\right)\;,b\left(t\right)\right)=\int_{0}^{t*}c^{T}\left(\tau \right)\Psi \left(t^{*}\;,\;\tau \right)b\left(\tau \right)b^{T}\left(\tau \right)\Psi^{T}\left(t^{*}\;,\;\tau \right)c\left(\tau \right)d\tau$

$G_{oc0\left[0,t*\right]}\left(c_{0}^{T},A_{0}\;,b_{0}\right)=\int_{0}^{t*}c_{0}^{T}e^{A_{0}\left(t^{*}-\tau \right)}b_{0}b_{0}^{T}e^{A_{0}^{T}\left(t^{*}-\tau \right)}c_{0}d\tau$ The (exact) point reachability of each system holds if the above respective scalar output controllability gramian on ${\left[0\;,\;t^{*}\right]}^{}$ is nonzero. Note that the nominal system is controllable implying point reachability for any time instant if and only if the output controllability matrix of the nominal linearized system ${\left[c_{0}^{T}\;,\;c_{0}^{T}A_{0}b_{0}\;,\;\cdots \;,c_{0}^{T}A_{0}^{n-1}b_{0}\right]}^{}\neq 0$. In the case of multi-output, i.e., $y\left(t\right)\in R^{p}$ with p≥2 and $c_{0}^{T}\to C_{0}$, $c^{T}\left(t\right)\to C\left(t\right)$, in the parameterizations and the controllability gramians, with $C_{0}\;,\;C\left(t\right)\in R^{p\times n}$ then the applicable condition is that the multi-output controllability gramian of the nominal, or respectively current, linearized system on ${\left[0\;,\;t^{*}\right]}^{}$ is nonsingular. For the nominal linearized system, the above property holds if and only if $rank{\left[C_{0}b_{0}\;,\;C_{0}A_{0}b_{0}\;,\;\cdots \;,C_{0}A_{0}^{n-1}b_{0}\;\right]}^{}=p$.

The results of the above sections can be extended directly for output reachability with minor direct changes.

The control laws for exact/approximate output targeting at the time instant $t^{*}>0$ are the subsequent ones for the case of zero input-output interconnections gains $d\left(t\right)\;,d_{0}$, or $D\left(t\right)\;,D_{0}$ if p≥2:

(a) For the exact reachability $\mathrm{OPR}\;\left(y^{*},t^{*}\;,x_{0}\right)$ of the current linearized linear system and also for the approximate reachability ($\mathrm{AOPR}\;\left(y^{*},t^{*}\;,x_{0}\right)$) of the current system at $t^{*}>0$ based on the same control, where $y^{*}=C\left(t^{*}\right)x^{*}$, the control law (37) is modified as follows:

(64) $$u\left(\tau \right)=b^{T}\left(\tau \right)\Psi^{T}\left(t^{*},\tau \right)G_{oc\left[0,t*\right]}^{-1}\left(C\left(\tau \right)\;,A\left(\tau \right)\;,b\left(\tau \right)\right)\left(x^{*}-\Psi \left(t^{*}\;,\;0\right)x_{0}\right);\;\tau \in {\left[0\;,\;t^{*}\right]}^{}$$

(b) For the $\mathrm{OPR}\;\left(y^{*},t^{*}\;,x_{0}\right)$ of the nominal linearized system and also for the $\mathrm{AOPR}\;\left(y^{*},t^{*}\;,x_{0}\right)$ of the current system based on the same control at $t^{*}>0$, the control law (12) is modified by replacing in (64):

$$b\left(\tau \right)\to b_{0},G_{oc\left[0,t*\right]}\left(C\left(\tau \right),A\left(\tau \right)\;,b\left(\tau \right)\right)\to G_{oc0\left[0,t*\right]}(C_{0},A_{0}\;,b_{0}).$$

### 4.2. Solvability Constraints When the Linearized Systems Are Not Controllable

If the linearized systems are non-controllable then the corresponding controllability gramians are singular. Therefore, the exact point reachability property introduces restrictions on the tentative targeted points $x^{*}$ at $t^{*}$ for the linearized systems for the given initial condition $x_{0}$. In particular, one has from (5) and the Rouché-Froebenius theorem from linear algebra, that for a control of the form;

(65) $$u_{0}\left(\tau \right)=b_{0}^{T}e^{A_{0}^{T}\left(t^{*}-\tau \right)}v_{0}\left(t^{*}\right)$$

guarantees the exact targeting condition:

(66) $$x^{*}-e^{A_{0}t^{*}}x_{0}-\int_{0}^{t^{+}}e^{A_{0}\left(t^{*}-\tau \right)}v_{x0}\left(\tau \right)d\tau =G_{c0\left[0,t*\right]}\left(A_{0},b_{0}\right)v_{0}\left(t^{*}\right)$$

for the current non-linear system under some existing auxiliary controls $v_{0}\left(t^{*}\right)$ if, for given $x_{0}$ and $t^{*}$,$x^{*}$ satisfies the constraint:

(67) $$rankG_{c0\left[0,t*\right]}\left(A_{0},b_{0}\right)=rank\left[G_{c0\left[0,t*\right]}\left(A_{0},b_{0}\right)\;,\;x^{*}-e^{A_{0}t^{*}}x_{0}-\int_{0}^{t^{*}}e^{A_{0}\left(t^{*}-\tau \right)}v_{x0}\left(\tau \right)d\tau \right]$$

The same control (65) has solutions for point reachability of the nominal linearized system if;

(68) $$rankG_{c0\left[0,t*\right]}\left(A_{0},b_{0}\right)=rank\left[G_{c0\left[0,t*\right]}\left(A_{0},b_{0}\right)\;,\;x^{*}-e^{A_{0}t^{*}}x_{0}\right]$$

**Proposition** **1.** *Assume that (68) holds for a given triple* $\left(x^{*},t^{*}\;,x_{0}\right)$ *. Then the nominal linearized system is* $\mathrm{PR}\;\left(x^{*},t^{*}\;,x_{0}\right)$ *and the current nonlinear system is* $\mathrm{PR}\;\left(x^{*},t^{*}\;,x_{0}\right)$ *for some targeted* ${\bar{x}}^{*}$ *which satisfies the following closeness to* $x^{*}$ *constraint:*

$$\left\|{\bar{x}}^{*}\right\|\in [\left|\left\|x^{*}\right\|-\left\|\int_{0}^{t^{*}}e^{A_{0}\left(t^{*}-\tau \right)}v_{x0}\left(\tau \right)d\tau \right\|\right|\;,\;\left\|x^{*}\right\|+\left\|\int_{0}^{t^{*}}e^{A_{0}\left(t^{*}-\tau \right)}v_{x0}\left(\tau \right)d\tau \right\|\;].$$

If the controllability gramian is singular but (68) holds then the algebraic system (66) is compatible indeterminate and has infinitely many solutions. Note that (68) also holds if the controllability gramian is non-singular. Therefore, (68) holds if and only if (66) is solvable. If (66) is solvable then Proposition 1 holds for approximate reachability of the current nonlinear system. It is also known [16] that, if (68) holds, then the either unique or the infinitely many solutions of (66) are found from the as:

(69) $$v_{0}\left(t^{*}\right)=G_{c0\left[0,t*\right]}^{†}\left(A_{0},b_{0}\right)\left(x^{*}-e^{A_{0}t^{*}}x_{0}-\int_{0}^{t^{+}}e^{A_{0}\left(t^{*}-\tau \right)}v_{x0}\left(\tau \right)d\tau \right)$$

where $G_{c0\left[0,t*\right]}^{†}\left(A_{0},b_{0}\right)$ is the Moore-Penrose pseudoinverse of $G_{c0\left[0,t*\right]}\left(A_{0},b_{0}\right)$, which coincides with the inverse if the controllability gramian is non-singular. If $G_{c0\left[0,t*\right]}\left(A_{0},b_{0}\right)$ has rank r(≤n) then it can be factorized as $G_{c0\left[0,t*\right]}\left(A_{0},b_{0}\right)=G_{C}G_{D}$, where $G_{C}\in R^{n\times r}$ and $G_{D}\in R^{r\times rn}$ are both of rank r. The subsequent result follows concerning all the set of solutions of (66), or the best approximated solution if (66) is algebraically incompatible, by taking into account the above considerations and the basic related results on pseudoinverse matrices in [15,16]:

**Theorem** **8.** *The following properties hold:*

*(i) Assume that (68) holds so that (66) is solvable in* $v_{0}\left(t^{*}\right)$ *. Then:*

(70) $$G_{c0\left[0,t*\right]}\left(A_{0},b_{0}\right)G_{c0\left[0,t*\right]}^{†}\left(A_{0},b_{0}\right)\left(x^{*}-e^{A_{0}t^{*}}x_{0}-\int_{0}^{t^{+}}e^{A_{0}\left(t^{*}-\tau \right)}v_{x0}\left(\tau \right)d\tau \right)=\left(x^{*}-e^{A_{0}t^{*}}x_{0}-\int_{0}^{t^{+}}e^{A_{0}\left(t^{*}-\tau \right)}v_{x0}\left(\tau \right)d\tau \right)$$

*and the control law (12) is calculated by the set of primary control solutions to the nominal linearized system is given by:*

(71) $$v_{0a}\left(t^{*}\right)=v_{0a}\left(t^{*}\;,\;V_{0}\right)=G_{c0\left[0,t*\right]}^{†}\left(A_{0},b_{0}\right)\left(x^{*}-e^{A_{0}t^{*}}x_{0}-\int_{0}^{t^{*}}e^{A_{0}\left(t^{*}-\tau \right)}v_{x0}\left(\tau \right)d\tau \right)\;+\left(I_{n}-G_{c0\left[0,t*\right]}^{†}\left(A_{0},b_{0}\right)G_{c0\left[0,t*\right]}\left(A_{0},b_{0}\right)\right)\;V_{0}$$

*where* $V_{0}\in R^{n}$ *is arbitrary and which becomes the unique solution (69) if the controllability gramian is non-singular.*

*(ii) Assume that (68) fails so that (66) is algebraically incompatible. Then, the primary control (71) with $V_{0}=0$ gives in the control law (12) the best approximation of the error norm for the reachability problem of the nominal linearized system in the sense that*

(72) $$v_{0a}\left(t^{*}\;,\;0\right)=Arg\min\;\left\|x^{*}-e^{A_{0}t^{*}}x_{0}-\int_{0}^{t^{+}}e^{A_{0}\left(t^{*}-\tau \right)}v_{x0}\left(\tau \right)d\tau -G_{c0\left[0,t*\right]}\left(A_{0},b_{0}\right)v_{0a}\left(t^{*}\;,\;V_{0}\right)\right\|$$

**Remark** **3.** *If the reachability of the current linearized system is taken as basis to solve the problem then (71) is replaced for an arbitrary $V_{c}\in R^{n}$ with;*

(73) $$v_{c}\left(t^{*}\right)=G_{c\left[0,t*\right]}^{†}\left(A\left(t\right),b\left(t\right)\right)\left(x^{*}-e^{A_{0}t^{*}}x_{0}-\int_{0}^{t^{*}}\Psi \left(t^{*},\;\tau \right)\xi \left(\tau \right)d\tau \right)\;+\left(I_{n}-G_{c\left[0,t*\right]}^{†}\left(A_{0},b_{0\left(A\left(t\right),b\left(t\right)\right)}\right)G_{c0\left[0,t*\right]}\left(A\left(t\right),b\left(t\right)\right)\right)\;V_{c}$$

*and a parallel result to Theorem 8 can easily by established for the two cases following when;*

(74) $$rankG_{c\left[0,t*\right]}\left(A\left(t\right),b\left(t\right)\right)=rank\left[G_{c\left[0,t*\right]}\left(A\left(t\right),b\left(t\right)\right)\;,\;x^{*}-e^{A_{0}t^{*}}x_{0}-\int_{0}^{t^{*}}\Psi \left(t^{*},\;\tau \right)\xi \left(\tau \right)d\tau d\tau \right]$$

*holds or fails by implementing the control law (37) based on the primary control (73).*

For the case of output reachability, one uses (64) with the output controllability gramian of the current linearized system for the counterpart of the control law (37) or its direct modification using the output controllability gramian of the nominal linearized system. In this way, we obtain either compatible controls or those giving the best approximation of the error norm if the problem is not solvable. Direct “ad hoc” extensions of Theorem 8 and Remark 3 are direct and are not detailed.

### 4.3. Constraints Associated with Saturated Controls

Assume that either (12) or (37), that is, the controls based on the linearized nominal or current systems are saturated to be constrained within prescribed closed domains. Then, 

a) Equation (12) is modified as follows:

(75) $$u_{0}\left(\tau \right)=sat_{u_{01},\;\;u_{02}}\left({\bar{u}}_{0}\left(\tau \right)\right)=\{\begin{array}{c} \;{\bar{u}}_{02}\;if\;{\bar{u}}_{0}\left(\tau \right)\geq {\bar{u}}_{02}\\ {\bar{u}}_{0}\left(\tau \right)\;if\;{\bar{u}}_{0}\left(\tau \right)\in \left({\bar{u}}_{01}\;,\;{\bar{u}}_{02}\right)\\ {\bar{u}}_{01}\;if\;{\bar{u}}_{0}\left(\tau \right)\leq {\bar{u}}_{01} \end{array};\;\tau \in {\left[0\;,\;t^{*}\right]}^{}$$

(76) $${\bar{u}}_{0}\left(\tau \right)=b_{0}^{T}e^{A_{0}^{T}\left(t^{*}-\tau \right)}G_{c\left[0,t*\right]}^{-1}\left(A_{0}\;,b_{0}\right)\left(x^{*}-e^{A_{0}t*}x_{0}\right);\;\tau \in {\left[0\;,\;t^{*}\right]}^{}$$

b) Equation (37) is modified as follows:

(77) $$u\left(\tau \right)=sat_{u_{1},\;\;u_{2}}\left(\bar{u}\left(\tau \right)\right)=\{\begin{array}{c} \;{\bar{u}}_{2}\;if\;{\bar{u}}_{}\left(\tau \right)\geq {\bar{u}}_{2}\\ \bar{u}\left(\tau \right)\;if\;\bar{u}\left(\tau \right)\in \left({\bar{u}}_{1}\;,\;{\bar{u}}_{2}\right)\\ {\bar{u}}_{1}\;if\;\bar{u}\left(\tau \right)\leq {\bar{u}}_{1} \end{array};\;\tau \in {\left[0\;,\;t^{*}\right]}^{}$$

(78) $$\bar{u}\left(\tau \right)=b^{T}\left(\tau \right)\Psi^{T}\left(t^{*},\tau \right)G_{c\left[0,t*\right]}^{-1}\left(A\left(\tau \right)\;,b\left(\tau \right)\right)\left(x^{*}-\Psi \left(t^{*}\;,\;0\right)x_{0}\right);\;\tau \in {\left[0\;,\;t^{*}\right]}^{}$$

The above modified saturated controls can be extended directly “mutatis-mutandis” to the subsequent problems:

1) Output reachability, for instance, the control effort (64) or its counterpart being based on the output reachability of the linearized nominal system. In this case, the targeting error for approximate reachability of Proposition 1 would become modified by including an error source generated by the deviation of the input from linearity as follows:
  (79) $$\left\|{\bar{x}}^{*}\right\|\in \left[\left|\left\|x^{*}\right\|-\left(\left\|\int_{0}^{t^{*}}e^{A_{0}\left(t^{*}-\tau \right)}\left(v_{x0}\left(\tau \right)+\left(\frac{{\bar{u}}_{01}}{sat_{{\bar{u}}_{01},\;{\bar{u}}_{02}}\left({\bar{u}}_{0}\left(\tau \right)\right)}-1\right)\right)d\tau \right\|+\right)\right|\;,\;\left\|x^{*}\right\|+\left\|\int_{0}^{t^{*}}e^{A_{0}\left(t^{*}-\tau \right)}\left(v_{x0}\left(\tau \right)+\left(1-\frac{{\bar{u}}_{02}}{sat_{{\bar{u}}_{01},\;{\bar{u}}_{02}}\left({\bar{u}}_{0}\left(\tau \right)\right)}\right)\right)d\tau \right\|\;\right]$$
  or the alternative expression derived under the reachability of the linearized current system.
2) Non-unique solvability or algebraic incompatibility as discussed in Theorem 8 and Remark 3 by adding similar error sources caused from the deviation of the input from linearity.

Typical examples of control saturation arise in vaccination in epidemic models since the vaccination effort cannot be negative and cannot be larger than unity if fractions of susceptible subpopulations are vaccinated via feedback.

## 5. Considerations on Reachability and Output Reachability in Some Epidemic Models Though Worked Examples

Some of the above concerns on reachability and approximate reachability are now discussed and emphasized on typical usual epidemic models which have in common the presence of nonlinear quadratic terms involving contributions to the dynamics of the products of susceptible and infectious subpopulations which plays a crucial role in the mechanism of the infective disease transmission. Such terms deviate the solution trajectory from the linear behavior about the equilibrium points. See, for instance, [5,6,7,8,9,10,11,12,13,14] and also [16,17,18,19,20,21] and some of the references therein.

**Example** **1.** *It can be argued that the epidemic models are not controllable or reachable in general. The following brief discussion leads to justify this claim. The so-called SEIR (including susceptible-exposed-infectious and recovered subpopulations) epidemic models possess typically a nonlinear quadratic term of the form βS(t)I(t), β being the disease coefficient transmission rate (which depends on the particular infectious disease under study), which governs the disease transmission. From biological considerations, all the state components (roughly speaking, the subpopulations of the model) have to be non-negative for all time. Assume an SEIR epidemic model with a unique disease-free equilibrium point and a unique endemic equilibrium point of a constant linear parameterization with linear vaccination effort*V(t)*whose state is*$x\left(t\right)={\left(S\left(t\right)\;,\;E\left(t\right)\;,\;I\left(t\right)\;,\;R\left(t\right)\right)}^{T}$*and whose total population*N(t)=S(t)+E(t)+I(t)+R(t)=N(0)*is constant for all time. This situation is common in many SEIR models. See, for instance, Reference [15]. Assume that the basic reproduction number [5,6], is less than unity so that the disease-free equilibrium point*$x_{e}=\left(S_{e},\;0\;,\;0\;,\;N\left(0\right)-S_{e}\right){}^{T}\neq 0$*is globally asymptotically stable,* [3,5,6]*. Since* $x\left(t\right)\to x_{e}$ *as* t→∞ *, it turns out that, for* $t^{*}=+\infty$ *, no other targeted state* $x^{*}\left(\neq x_{e}\right)$ *can be prefixed as objective for any given initial state* x(0) *even for the current linearized version. As a result,* $G_{oc\left[0,\infty \right)}\left(A_{0}\;,b_{0}\right)=\underset{t^{*}\to \infty }{\lim}\int_{0}^{t*}e^{A_{0}\left(t^{*}-\tau \right)}b_{0}b_{0}^{T}e^{A_{0}^{T}\left(t^{*}-\tau \right)}d\tau$ *is singular and the linearized system is not asymptotically controllable and it is not asymptotically point-reachable for arbitrarily fixed* $x\left(\infty \right)=x^{*}\neq x_{e}$ $t^{*}=+\infty$*. Since the integrand of the gramian is a semidefinite matrix, so that all its eigenvalues are non-negative and at least one of them is positive. Note, by inspection, that the maximum eigenvalue of the integrand* $\lambda_{\max}G_{oc\left[0,\infty \right)}\left(A_{0}\;,b_{0}\right)\left(\tau \right)=e^{A_{0}\left(t^{*}-\tau \right)}b_{0}b_{0}^{T}e^{A_{0}^{T}\left(t^{*}-\tau \right)}>0$ *if* $0\leq t^{*}-\tau <+\infty$ *, that is,* $\underset{t^{*}\to \infty }{\lim}b_{0}^{T}e^{A_{0}^{T}t^{*}}\left(\int_{0}^{\infty }e^{-A_{0}^{T}\tau }e^{-A_{0}\tau }d\tau \right)\;e^{A_{0}t^{*}}b_{0}$*) is positive.*

Since $G_{oc\left[0,\infty \right)}\left(A_{0}\;,b_{0}\right)=\underset{t^{*}\to \infty }{\lim}\int_{0}^{t*}e^{A_{0}\left(t^{*}-\tau \right)}b_{0}b_{0}^{T}e^{A_{0}^{T}\left(t^{*}-\tau \right)}d\tau$ is singular then its maximum eigenvalue is infinity, that is, $\underset{t^{*}\to \infty }{\lim}b_{0}^{T}e^{A_{0}^{T}t^{*}}\left(\int_{0}^{\infty }e^{-A_{0}^{T}\tau }e^{-A_{0}\tau }d\tau \right)\;e^{A_{0}t^{*}}b_{0}=+\infty$ and the maximum eigenvalue of the gramian. It can be said that is it asymptotically reachable from any initial condition if the targeted state at infinity only if the disease-free equilibrium point, i.e., $x^{*}\left(\infty \right)=x_{e}$. From Definition 2, the nominal linearized system is not reachable either since reachability fails at infinite time for any point except for the disease- free equilibrium one. However, it can be point reachable for certain given triples $\left(x^{*}\;,t^{*},x_{0}\right)$ which should be necessarily subject to the constraint the that the sum of their components equalize N(0). Point reachability is not possible at the time instant $t^{*}$ if such a constraint is violated. The same conclusion can arise for the current linearized system. Just from the above empiric consideration on necessary conditions for reachability, we can conclude that:

Asymptotic reachability of both the nominal linearized and current system are only achievable if the targeted point at infinity time is the disease-free equilibrium point. In particular, the exposed and infectious subpopulations should be zero. The only freedom is that such a point can be governed by the steady-state vaccination effort which allows to modify correspondingly, depending on such an effort, the equilibrium susceptible and recovered subpopulations while keeping each component non-negative and their sum equal to the total population.

Finite-time reachability at the time instant $t^{*}$ of the linearized system about the disease-free equilibrium point is only achievable if the targeted state has non-negative components whose sum equalizes the initial total population. Since there is no reachability of the nominal linearized system for arbitrary triples of initial conditions, targeted state and targeted time, one concludes that the pair $\left(A_{0}\;,\;b_{0}\right)$ of such SEIR epidemic models is not controllable via vaccination controls.

If the reachability of the linearized systems in the sense of Definition 2 for an arbitrary targeted point about the equilibrium fail then that of the current system also fails. The same above basic principles are kept for the asymptotic reachability of the endemic equilibrium point in the case when the reproduction number exceeds unity.

If the problem is stated for output-reachability with the dimension of the output less than that of the state (for instance, the output has only one to three of the sate components) then the considerations are close but the constraints are easy to satisfy. For instance, the targeted output has only to be constrained to its components to be non-negative and their sum to be less than or equal to the initial total population.

A simple intuitive entropy-based interpretation of the probabilities of both attractors to be the relevant equilibrium point is as follows. Note that we cope with a very common situation that the epidemic model possess a unique disease-free equilibrium point and a unique endemic one. Then, if the reproduction number $R_{0}\leq 1$ (typically, the disease transmission rate β does not exceed a certain critical value $\beta_{c}$ associated with $R_{0}=1$) then the endemic equilibrium point typically does not exist as being compatible with the non-negative solution trajectories while the unique globally asymptotically stable attractor is the disease-free one. So, we can say that that the probability of the trajectory to reach the first one is $p_{df}=1$ while that of reaching the second one is $p_{end}=0$. Thus, the entropy is $H=-\left(p_{df}\ln p_{df}+p_{end}\ln p_{end}\right)=0$ [22]. The same conclusion arises if $R_{0}>1$ (typically, the transmission rate β exceeds the critical value $\beta_{c}$) since then the disease-free equilibrium point is unstable while the endemic one is asymptotically stable $p_{df}=0$ and $p_{end}=1$ and again H=0. In general, if the reproduction number lies in $\left[1-\gamma_{1}\;,\;1+\gamma_{2}\right]$, with $\gamma_{1}\in \left[0\;,\;1\right]$ and $\gamma_{2}\geq 0$, or if the transmission rate can oscillate around the critical value, that is $\beta \in \left[\beta_{c}-\delta_{1}\;,\;\beta_{c}+\delta_{2}\right]$, then $p_{df}=\alpha \in \left[0\;,\;1\right]$, $p_{end}=\left(1-\alpha \right)\in \left[0\;,\;1\right]$ and the entropy is H(α)=−(αlnα+(1−α)ln(1−α)). As a result if α is close to unity (respectively, to zero) then the disease-free equilibrium (respectively, the endemic equilibrium point) is the “most probable” attractor. In particular, H(0)=H(1)=0, that is the solution trajectory converges either to the disease-free equilibrium point $\alpha =\gamma_{1}=1$ and $\delta_{1}\in \left[0\;,\;\beta_{c}\right]$, $\gamma_{2}=\delta_{2}=0$) or to the endemic one ($\alpha =\gamma_{1}=\delta_{1}=0$ and $\gamma_{2}>0$, $\delta_{2}>0$), and $\underset{\alpha \in \left[0\;,\;1\right]}{\max}H\left(\alpha \right)=H\left(1/2\right)=-\ln\left(1/2\right)=0.6931>0$ gives the maximum uncertainty about which equilibrium is the most probable attractor indicating that both of them are “unlikely probable with the same uncertainty degree”.

**Example** **2.** *Consider the subsequent SIR model with time-invariant parameterization and a vaccination control*V(t)*including an additive term proportional to the susceptible and another eventual free-choice additive term:*

(80) $$\dot{S}\left(t\right)=-\beta I\left(t\right)S\left(t\right)-V\left(t\right)=-\left(\beta I\left(t\right)+K_{V}\right)S\left(t\right)-g\left(t\right)\;\dot{I}\left(t\right)=\left(\beta S\left(t\right)-\nu \right)I\left(t\right)\;\dot{R}\left(t\right)=\nu I\left(t\right)+V\left(t\right)=K_{V}S\left(t\right)+vI\left(t\right)+g\left(t\right)\;V\left(t\right)=K_{V}S\left(t\right)+g\left(t\right)$$

*where*β*is the disease transmission rate and*ν*is the removal rate,*$K_{V}\in \left[0\;,\;1\right]$*is the control gain of vaccination of a fraction of the susceptible and*$\left(-K_{V}S\left(t\right)\leq \right)g\left(t\right)\to g_{e}\left(\geq 0\right)$*as*t→∞*. The total population is*N(t)=S(t)+I(t)+R(t) By summing up the three first equations one gets that the resulting right-hand-side is identically zero $\dot{N}\left(t\right)=0$ *so that the total population* N(t)=N(0)=S(0)+I(0)+R(0) *for all time. The equilibrium points are* $x_{e}={\left(S_{e}\;,\;I_{e},R_{e}\right)}^{T}$ *such that the three above time-derivatives are zero. So, the algebraic equation of the equilibrium points is:*

$$\left[\begin{array}{ccc} -\left(\beta I_{e}+K_{V}\right) & 0 & 0\\ 0 & \beta S_{e}-\nu & 0\\ K_{V} & \nu & 0 \end{array}\right]\;x_{e}=\left[\begin{array}{c} g_{e}\\ 0\\ -g_{e} \end{array}\right]=\left[\begin{array}{c} 1\\ 0\\ -1 \end{array}\right]g_{e}$$

*where the coefficient matrix is the Jacobian matrix corresponding to such an equilibrium. Thus, one has the subsequent cases:*

*1) If* g(t)≥0 *so that* $g_{e}\geq 0$ *then:*

$$S_{e}=-\frac{g_{e}}{\beta I_{e}+K_{V}}\geq 0\Rightarrow S_{e}=g_{e}=0$$

$$\left(\beta S_{e}-\nu \right)I_{e}=-\nu I_{e}=0\Rightarrow I_{e}=0$$

$$K_{V}S_{e}+\nu I_{e}=-g_{e}=0\mathrm{for}\mathrm{any}K_{V}\geq 0$$

$$R_{e}=N\left(0\right)-S_{e}-I_{e}=N\left(0\right)$$

$$V_{e}=\underset{t\to \infty }{\lim}V\left(t\right)=K_{V}S_{e}+g_{e}=0\mathrm{for}\mathrm{any}K_{V}\in \left[0\;,\;1\right]$$

*Then, the unique equilibrium point is a disease-free one* $x_{e}={\left(0\;,\;0\;,\;N\left(0\right)\right)}^{T}$ *, which depends on the initial conditions, and with the whole population being asymptotically immune. The spectrum of the Jacobian matrix* $J=J\left(x_{e}\right)$ *is* $\Lambda =\left\{0\;,-K_{V},-\nu \right\}$ *which is critically stable with two zero eigenvalues if* $K_{V}\neq 0$ *and one critically stable eigenvalue if* $K_{V}=0$ *, so that, in the absence of vaccination the stability properties become worsened with respect to the application of proportional vaccination to the susceptible.*

*2) If*g(t)≤0*, so that*$g_{e}\leq 0$*, with* |*g*(*t*)| ≤ *K_V_S*(*t*) ≤ *S*(*t*) *guaranteeing that* V(t)≥0 *then*

$S_{e}=\frac{\left|g_{e}\right|}{\beta I_{e}+K_{V}}\geq 0$ *, and*

$\left(\beta S_{e}-\nu \right)I_{e}=\left(\frac{\beta \left|g_{e}\right|}{\beta I_{e}+K_{V}}-\nu \right)I_{e}=0\Rightarrow I_{e}=0$*or $I_{e}=\frac{\beta \left|g_{e}\right|-\nu K_{V}}{\beta \nu }$ which is zero (disease-free equilibrium point) if*$\left|g_{e}\right|=\frac{\nu \;K_{V}}{\beta }$*and nonzero (endemic equilibrium point) if*$\left|g_{e}\right|=\left(\beta I_{e}+K_{V}\right)S_{e}>\frac{\nu \;K_{V}}{\beta }$*, that is, if*$S_{e}>\frac{\nu K_{V}}{\beta \left(\beta I_{e}+K_{V}\right)}$.

$K_{V}S_{e}+\nu I_{e}=-g_{e}=0$*for any*$K_{V}\geq 0$*what implies that either*$K_{V}S_{e}=\nu I_{e}=\left|g_{e}\right|=0$*, so that*$S_{e}=I_{e}=g_{e}=0$*, or*$K_{V}=I_{e}=g_{e}=0$*. As a result for both case of*$K_{V}$*being zero or non-zero, the endemic equilibrium point does not exist since it would make incompatible the conditions associated with the second row of the Jacobian matrix. Since*$g_{e}=0$*then*$S_{e}=\frac{\left|g_{e}\right|}{\beta I_{e}+K_{V}}=0$*irrespective of the vaccination control gain*$K_{V}$*being zero or nonzero. Again,*$R_{e}=N\left(0\right)-S_{e}-I_{e}=N\left(0\right)$*and*$V_{e}=\underset{t\to \infty }{\lim}V\left(t\right)=K_{V}S_{e}+g_{e}=0$*for any*$K_{V}\in \left[0\;,\;1\right]$. *As a result, the unique feasible equilibrium point is the disease-free one*$x_{e}$*of Case 1 which is again critically stable.*

By considering the spectrum of the Jacobian matrix, it is seen that there are a critically stable eigenvalue and a stable eigenvalue which cannot be either removed or prefixed and that the other one can be prefixed by the choice of the control gain. Then, note the following facts:

It is obvious that the pair $\left(A_{0}\;,\;b_{0}\right)$ with $A_{0}=J$ for $S_{e}=I_{e}=0$, $R_{e}=N\left(0\right)$ and $b_{0}={\left(-1\;,\;0\;,\;1\right)}^{T}$ is neither controllable nor stabilizable so that the nominal linearized system is not reachable in the sense of Definition 2 for any given arbitrary triple $\left(x^{*},t^{*}\;,x_{0}\right)$. In particular, note that $rank\left(b_{0}\;,\;Ab_{0},A^{2}b_{0}\right)=1<3$. This implies that the linearized nominal system cannot be reachable at any finite time for any arbitrary targeted state in the sense of Definition 2. 

The linearized nominal system is exactly output-reachable if the output is defined to be the susceptible subpopulation and the vaccination has no maximum constraint. Assume that the vaccination control effort is just the proportional term to the susceptible, i.e., g(t)≡0. Since the susceptible and the infectious at the equilibrium are both zero, one has that the susceptible of the linearized nominal system are $S\left(t\right)=e^{-K_{V}t^{*}}S_{0}$. Assume that the targeted susceptible are $S^{*}<S_{0}$ at some $t^{*}>0$. Then, $K_{V}=K_{V}^{*}=-\frac{\ln S^{*}}{t^{*}\ln S_{0}}$ targets $S\left(t^{*}\right)=S^{*}<S_{0}$. However, if $S^{*}\geq S_{0}$ then $K_{V}\leq 0$ and the (exact) point reachability of the linearized nominal system is unfeasible since vaccination cannot be implemented with negative gains which would lead to increase the susceptible numbers.

In practice, the vaccination gain is restricted to $K_{V}\in \left[0\;,\;1\right]$ since the vaccination decreases the number of susceptible and one cannot vaccinate more individuals that the susceptible amounts at each time. Therefore, $K_{V}\in \;\left[0\;,\;\max\left(1,\;\;-\frac{\ln S^{*}}{t^{*}\ln S_{0}}\right)\right]$ for a given $t^{*}$.

The current system is approximately reachable with a certain targeting error at any time instant $t^{*}>0$ if the vaccination control is unconstrained. In fact, $\dot{S}\left(t\right)=-\left(\beta I\left(t\right)+K_{V}^{*}\right)S\left(t\right)$ so that if $K_{V}=K_{V}^{*}=-\frac{\ln S^{*}}{t^{*}\ln S_{0}}>0$ has no upper-bound constraint then one gets:

(81) $$S\left(t^{*}\right)=e^{-\int_{0}^{t}\left(\beta I\left(\tau \right)+K_{V}d\tau \right)}S_{0}=e^{-\int_{0}^{t^{*}}\beta I\left(\tau \right)d\tau }\left(e^{-K_{V}t^{*}}S_{0}\right)=e^{-\int_{0}^{t^{*}}\beta I\left(\tau \right)d\tau }S^{*}\;=e^{-I_{0}\int_{0}^{t^{*}}\beta e^{-\nu \tau }d\tau }S^{*}=e^{-\beta I_{0}\left(1-e^{-\nu t^{*}}\right)/\nu }S^{*}$$

and, if $S^{*}=S_{0}e^{\lambda_{S}^{*}}$ for some real $\lambda_{S}^{*}<0$, then the error between the susceptible of the current system at the targeting time instant and the targeted susceptible of the linearized nominal systems is:

(82) $$\left|\ln S\left(t^{*}\right)-\ln S^{*}\right|=\beta I_{0}\left(1-e^{\frac{\nu \left(\ln S_{0}-\left|\lambda_{S}^{*}\right|\right)}{K_{V}\ln S_{0}}}\right)/\nu =\beta I_{0}\left(1-e^{\frac{\nu \left(\ln S_{0}-\left|\lambda_{S}^{*}\right|\right)}{K_{V}\ln S_{0}}}\right)/\nu$$

However, if the vaccination control is constrained as $K_{V}\in \;{\left[0\;,\;\max\left({\bar{K}}_{V},\;\;K_{V}^{*}\right)\right]}^{}$ with $K_{V}^{*}=-\frac{\ln S^{*}}{t^{*}\ln S_{0}}>{\bar{K}}_{V}$ for some given ${\bar{K}}_{V}\leq 1$, which establishes the maximum fraction of susceptible allowed to be vaccinated, then $K_{V}={\bar{K}}_{V}=K_{V^{*}}-\left|{\bar{K}}_{V}-K_{V^{*}}\right|$, and:

(83) $$S\left(t^{*}\right)=e^{-\int_{0}^{t}\left(\beta I\left(\tau \right)+\left(K_{V^{*}}-\left|{\bar{K}}_{V}-K_{V^{*}}\right|\right)d\tau \right)}S_{0}=e^{-\int_{0}^{t^{*}}\beta I\left(\tau \right)d\tau }\left(e^{-K_{V^{*}}t^{*}}S_{0}\right)\;\left(e^{-\left|{\bar{K}}_{V}-K_{V^{*}}\right|t^{*}}\right)\;=e^{-\left[\beta I_{0}\left(1-e^{-\nu t^{*}}\right)/\nu +\left|{\bar{K}}_{V}-K_{V^{*}}\right|t^{*}\right]}S^{*}$$

Thus, (82) becomes modified as follows:

(84) $$\left|\ln S\left(t^{*}\right)-\ln S^{*}\right|=\beta I_{0}\left(1-e^{\frac{\nu \left(\ln S_{0}-\left|\lambda_{S}^{*}\right|\right)}{K_{V}^{*}\ln S_{0}}}\right)/\nu +\left|{\bar{K}}_{V}-K_{V^{*}}\right|t^{*}$$

if $t^{*}<\frac{\left|\lambda_{S}^{*}\right|}{\ln S_{0}}-1$ since $K_{V}^{*}>{\bar{K}}_{V}$ and $K_{V}={\bar{K}}_{V}<K_{V}^{*}$, and:

(85) $$\left|\ln S\left(t^{*}\right)-\ln S^{*}\right|=\beta I_{0}\left(1-e^{\frac{\nu \left(\ln S_{0}-\left|\lambda_{S}^{*}\right|\right)}{K_{V}\ln S_{0}}}\right)/\nu =\beta I_{0}\left(1-e^{\frac{\nu \left(\ln S_{0}-\left|\lambda_{S}^{*}\right|\right)}{K_{V}\ln S_{0}}}\right)/\nu$$

if $t^{*}\geq \frac{\left|\lambda_{S}^{*}\right|}{\ln S_{0}}-1$ since then $K_{V}=K_{V}^{*}$.

**Example** **3.** *Consider the following* SI *epidemic model with* N *interacting groups and the infection being transmitted within and between groups which includes vaccination efforts and which was proposed (in the vaccination-free case) in [17,18]:*

(86) $${\dot{I}}_{j}\left(t\right)=\beta \left(N_{j}-I_{j}\left(t\right)\right)\left(\beta_{jr}I_{j}\left(t\right)+\sum_{i\left(\neq j\right)=1}^{N}\beta_{ijr}I_{i}\left(t\right)\right)-T_{j}\left(t\right)\;\;j=1,2,\ldots ,N$$

*subject to initial conditions* $I_{j}\left(0\right)=I_{j0}$ *;* j=1,2,…,N *, where* $N_{j}\;,I_{j}$ *and* $S_{j}=N_{j}-I_{j}$ *;* j=1,2,…,N *are the total, infectious and susceptible populations of the* N *groups with respective total populations at each group being constant: and where* $\beta_{ij}$ *;* i,j=1,2,…,N *is the mutual disease coefficient transmission rate from the* i−th *to the* j−th *group,* β *is a reference disease coefficient transmission rate (typically, either the minimum, or the maximum or an average amount,* $\beta_{ijr}=\beta_{ij}/\beta$ *;* i,j=1,2,…,N *, with* $\beta_{jj}=\beta_{j}$ *and* $\beta_{jjr}=\beta_{jr}$ *being a simplified notation for any* j=1,2,…,N *, are the relative values of the* $\beta_{ij}$ *related to* β *and* $T_{j}$ *;* j=1,2,…,N *are the antiviral treatment effort son the infectious. Assume that:*

(87) $$T_{j}\left(t\right)=K_{j}\left(t\right)\left(N_{j}-I_{j}\left(t\right)\right)\;\;K_{j}\left(t\right)\;=\sum_{i=1}^{N}\beta_{ij}I_{i}\left(t\right)+\varepsilon_{j}\left(t\right);j=1,2,\ldots ,N$$

*for any* $\varepsilon_{j}:\left[0\;,\;\infty \right)\to {\left[0\;,\;\varepsilon_{j1}\right)}^{}$ *, with* $\varepsilon_{j}\left(t\right)=0$ *if and only if* $I_{j}\left(t\right)=0$ *;* j=1,2,…,N *. But* $K_{j}\left(t\right)\in \left[0,\;1\right]$ *, implies that*

(88) $$\varepsilon_{j}\left(t\right)\leq \varepsilon_{j1}\leq 1-\sum_{i=1}^{N}\beta_{ij}I_{i}\left(0\right)\leq 1-\sum_{i=1}^{N}\beta_{ij}I_{i}\left(t\right);j=1,2,\ldots ,N$$

*since* $K_{j}\left(t\right)\left(N_{j}-I_{j}\left(t\right)\right)$ *is the fraction of susceptible used for antiviral treatment of the infectious at time* t *;* j=1,2,…,N *. Thus, it suffices that* $\sum_{i=1}^{N}\beta_{ij}I_{i}\left(0\right)\leq 1-\varepsilon_{j1}$ *and* $\varepsilon_{j1}<1$ *;* j=1,2,…,N *for (88) to hold resulting in* $I_{j}\left(t\right)$ *being strictly monotonically decreasing, then* $I_{j}\left(t\right)\to 0$ *as* t→∞ *;* j=1,2,…,N *It has been proved the following:*

**Proposition** **2.** *Assume that the epidemic model (86) is subject to the treatment control law (87) for any* $\varepsilon_{j}:\left[0\;,\;\infty \right)\to {\left[0\;,\;\varepsilon_{j1}\right)}^{}$ *, with* $\varepsilon_{j}\left(t\right)=0$ *if and only if* $I_{j}\left(t\right)=0$ *;* j=1,2,…,N *, under the constraints* $\varepsilon_{j1}<1$ *and sufficiently small initial conditions such that* $\sum_{i=1}^{N}\beta_{ij}I_{i}\left(0\right)\leq 1-\varepsilon_{j1}$ *. Then, all the infectious subpopulations converge strictly monotonically to zero so that the susceptible subpopulations of each group converge monotonically to the total subpopulations of the corresponding groups. Furthermore, all the subpopulations are non-negative for all time under non-negative initial conditions.*

Assume now that the antiviral control (87) is modified as follows:

(89) $$T_{j}\left(t\right)=\sum_{i=1}^{N}\lambda_{ij}\left(t\right)I_{i}\left(t\right)I_{j}\left(t\right)$$

where $\lambda_{ij}:\left[0\;,\;\infty \right)\to \left[0\;,\;\infty \right)$; i,j=1,2,…,N. The replacement of (89) into (86) yields:

(90) $${\dot{I}}_{j}\left(t\right)=\sum_{i=1}^{N}\left[\beta_{ij}N_{j}-\left(\beta_{ij}+\lambda_{ij}\left(t\right)\right)I_{i}\left(t\right)\right]I_{j}\left(t\right)\;;\;j=1,2,\ldots ,N$$

Now, choose:

(91) $$\lambda_{ij}\left(t\right)=\frac{1}{I_{i}\left(t\right)}\left(\varepsilon_{ij}\left(t\right)+\beta_{ij}\left(N_{j}-I_{j}\left(t\right)\right)\right)$$

if $I_{i}\left(t\right)\neq 0$ and $\lambda_{ij}\left(t\right)=0$ if $I_{i}\left(t\right)=0$; i,j=1,2,…,N for some $\varepsilon_{ij}:\left[0\;,\;\infty \right)\to \left[0\;,\;\infty \right)$; i,j=1,2,…,N, subject to $\sum_{i=1}^{N}\lambda_{ij}\left(t\right)I_{i}\left(t\right)\leq 1$ for all t≥0, j=1,2,…,N so that (89) consists of giving a treatment on a fraction of the infectious of that j-th group. This implies the subsequent constraint:

(92) $$\sum_{i=1}^{N}\lambda_{ij}\left(t\right)\left(\varepsilon_{ij}\left(t\right)+\beta_{ij}\left(N_{j}-I_{j}\left(t\right)\right)\right)\leq 1$$

which is guaranteed if $\lambda_{ij}\left(t\right)\in \left[\;0\;,\;\frac{1}{N\;{\left(\varepsilon_{ij}\left(t\right)+\beta_{ij}\left(N_{j}-I_{j}\left(t\right)\right)\right)}^{}}\right]$; i,j=1,2,…,N. Under (92), one has from (90) that ${\dot{I}}_{j}\left(t\right)=-\sum_{i=1}^{N}\varepsilon_{ij}\left(t\right)$ so that $I_{j}\left(t\right)\leq I_{j}\left(0\right)$ for all t≥0 according to:

(93) $$I_{j}\left(t\right)=e^{-\sum_{i=1}^{N}\int_{0}^{t}\varepsilon_{ij}\left(\tau \right)d\tau }I_{j}\left(0\right)=e^{-\sum_{i=1}^{N}\int_{0}^{t}\left(\lambda_{ij}\left(\tau \right)I_{i}\left(\tau \right)-\beta_{ij}\left(N_{j}-I_{j}\left(\tau \right)\right)\right)d\tau }I_{0}\;;\;j=1,2,\ldots ,N$$

with $I_{j}\left(0\right)=I_{j0}$; j=1,2,…,N. Then the reachability for any suited targeted state in the sense of Definition 2 is not possible. However, the current system is $\mathrm{PR}\;\left({\bar{I}}^{*},t^{*}\;,{\bar{I}}_{0}\right)$ where ${\bar{I}}^{*}={\left(I_{1}^{*},\;I_{2}^{*},\;\cdots \;I_{N}^{*}\right)}^{T}$ and ${\bar{I}}_{0}={\left(I_{10}\;,\;I_{20}\;,\cdots \;,\;I_{N0}\right)}^{T}$ with the N constraints $I_{j}^{*}\geq I_{j0}$;j=1,2,…,N, if $\varepsilon_{ij}\left(t\right)$; i,j=1,2,…,N are such that the control gains $\lambda_{ij}:\left[0\;,\;\infty \right)\to \left[0\;,\;\infty \right)$; i,j=1,2,…,N are selected in (91) for a set of functions $\varepsilon_{ij}\left(t\right)$ such that $\rho_{j}=\sum_{i=1}^{N}\int_{0}^{t^{*}}\varepsilon_{ij}\left(\tau \right)d\tau /t^{*}=-\frac{1}{t^{*}}\ln\left(\frac{I_{j}^{*}}{I_{j0}}\right)$; j=1,2,…,N.

## Appendix A. Auxiliary Technical Results

**Lemma** **A1.**
$F\left(t\right)=I_{n}-\int_{0}^{t}e^{A_{0}\left(t-\tau \right)}\tilde{A}\left(\tau \right)\Psi \left(\tau ,t\right)d\tau$ *is non-singular for a given* $t\in R_{+}$ *if the following two assumptions hold:*
*1)*$A_{0}$*is a stability matrix so that*$\left\|e^{A_{0}t}\right\|\leq K_{0}e^{-\rho_{0}t}$*;*$\forall t\in R_{0+}$*for some real constants*$K_{0}\left(\geq 1\right)$*and*$\rho_{0}>0$,
*2)*

(A1) $$\left\|\int_{0}^{t}e^{A_{0}\left(t-\tau \right)}\tilde{A}\left(\tau \right)\Psi \left(\tau ,t\right)d\tau \right\|<1$$

*and (A1) holds if the assumption 3 below holds:*

*3)*

(A2) $$\underset{0\leq \tau \leq t}{\sup}\left\|\tilde{A}\left(\tau \right)\right\|<1/\left\|\int_{0}^{t}e^{A_{0}\left(t-\tau \right)}\Psi \left(\tau ,t\right)d\tau \right\|$$

*and (A2) holds if the assumption 4 below holds:*

*4)*

(A3) $$\underset{0\leq \tau \leq t}{\sup}\left\|\tilde{A}\left(\tau \right)\right\|<\rho_{0}/K_{0}\left(1-e^{-\rho_{0}t}\right)\left\|\Psi \left(\tau ,t\right)d\tau \right\|$$

*where* Ψ(t,τ) *;* $\forall t\left(\geq \tau \right),\;\tau \in R_{0+}$ *, which satisfies* $\left\|\Psi \left(t,\tau \right)\right\|\leq K_{\Psi }e^{-\rho_{\Psi }\left(t-\tau \right)}$ *;* $\forall t\left(\geq \tau \right),\;\tau \in R_{0+}$ *,is the fundamental* *of the system* $\dot{x}\left(t\right)=A\left(t\right)x\left(t\right)$ *,* $x\left(0\right)=x_{0}$ *, and (A3) holds if the assumption 5 below holds,*
*5)*

(A4) $$\underset{0\leq \tau \leq t}{\sup}\left\|\tilde{A}\left(\tau \right)\right\|<\left(\rho_{0}-\rho_{\Psi }\right)/K_{0}K_{\Psi }\left(1-e^{-\left(\rho_{0}-\rho_{\Psi }\right)\;t}\right)$$

*for some**real constants*$K_{\Psi }\geq 1$*and*$\rho_{\Psi }\geq \rho_{0}-\underset{0\leq t<+\infty }{\sup}\left\|\tilde{A}\left(t\right)\right\|>0$*provided that*$\underset{\tau \leq \sigma \leq t}{\sup}\left\|\tilde{A}\left(\sigma \right)\right\|<\frac{\rho_{0}}{K_{0}}$*;*∀t(≥τ), τ∈R*and that*$\rho_{\Psi }\leq \rho_{0}\leq \rho_{\Psi }+\underset{0\leq t<+\infty }{\sup}\left\|\tilde{A}\left(t\right)\right\|$.

**Proof** Note that if Ψ(t,τ) is the fundamental matrix associated with A(t). Then,

(A5) $$\Psi \left(t,\tau \right)=e^{A_{0}\left(t-\tau \right)}+\int_{\tau }^{t}e^{A_{0}\left(t-\sigma \right)}\tilde{A}\left(\sigma \right)\Psi \left(t,\sigma \right)d\sigma \;;\;\forall t\left(\geq \tau \right),\tau \in R_{0+}$$

with $\Psi \left(\tau ,\tau \right)=I_{n}$; $\forall \tau \in R_{0+}$, which is of exponential order on the interval τ≤σ≤t for any t(≥τ), τ so that there exist real constants $K_{\Psi }\geq 1,\rho_{\Psi }\in R$ such that $\left\|\Psi \left(t,\tau \right)\right\|\leq K_{\Psi }e^{-\rho_{\Psi }\left(t-\tau \right)}$; $\forall t\left(\geq \tau \right),\tau \in R_{0+}$. On the other hand, note that

(A6) $$\left\|\Psi \left(t,\tau \right)\right\|\leq K_{0}e^{-\rho_{0}\left(t-\tau \right)}+\frac{K_{0}}{\rho_{0}}\left(1-e^{-\rho_{0}\left(t-\tau \right)}\right)\underset{\tau \leq \sigma \leq t}{\sup}\left\|\tilde{A}\left(\sigma \right)\right\|\underset{\tau \leq \sigma \leq t}{\sup}\left\|\Psi \left(t,\sigma \right)\right\|;\;\forall t\left(\geq \tau \right),\;\tau \in R_{0+}$$

Note from Banach´s Perturbation Lemma and (A6) that one has for any $t\in R_{0+}$:

(A7) $${\left\|F\left(t\right)\right\|}^{-1}\leq \varepsilon_{{}_{5FM}}\left(t\right)=\frac{\rho_{0}-\rho_{\Psi }}{\rho_{0}-\rho_{\Psi }-K_{0}K_{\Psi }{\left(1-e^{-\left(\rho_{0}-\rho_{\Psi }\right)\;t}\right)}^{}\underset{0\leq \tau \leq t}{\sup}\left\|\tilde{A}\left(\tau \right)\right\|}\;(\mathrm{if}\;(A4)\;\mathrm{holds})$$

(A8) $$\leq \varepsilon_{{}_{4FM}}\left(t\right)=\frac{\rho_{0}}{\rho_{0}-K_{0}{\left(1-e^{-\rho_{0}t}\right)}^{}\left\|\int_{0}^{t}\Psi \left(\tau ,t\right)d\tau \right\|\underset{0\leq \tau \leq t}{\sup}\left\|\tilde{A}\left(\tau \right)\right\|}\;(\mathrm{if}\;(A3)\;\mathrm{holds})$$

(A9) $$\leq \varepsilon_{{}_{3FM}}\left(t\right)=\frac{1}{1-\left\|\int_{0}^{t}e^{A_{0}\left(t-\tau \right)}\Psi \left(\tau ,t\right)d\tau \right\|\underset{0\leq \tau \leq t}{\sup}\left\|\tilde{A}\left(\tau \right)\right\|}\;(\mathrm{if}\;(A2)\;\mathrm{holds})$$

(A10) $$\leq \varepsilon_{{}_{2FM}}\left(t\right)=\frac{1}{1-\left\|\int_{0}^{t}e^{A_{0}\left(t-\tau \right)}\tilde{A}\left(\tau \right)\Psi \left(\tau ,t\right)d\tau \right\|}\;(\mathrm{if}\;(A1)\;\mathrm{holds})$$

□

**Lemma** **A2.**
*One has that:*

(A11) $${\left\|F\left(t\right)\right\|}^{-1}\geq \varepsilon_{{}_{5Fm}}\left(t\right)=\frac{\rho_{0}-\rho_{\Psi }}{\rho_{0}-\rho_{\Psi }+K_{0}K_{\Psi }{\left(1-e^{-\left(\rho_{0}-\rho_{\Psi }\right)\;t}\right)}^{}\underset{0\leq \tau \leq t}{\sup}\left\|\tilde{A}\left(\tau \right)\right\|}$$

*if the assumptions 1 and 5 of Lemma A2 hold:*

(A12) $${\left\|F\left(t\right)\right\|}^{-1}\geq \varepsilon_{{}_{4Fm}}\left(t\right)=\frac{\rho_{0}}{\rho_{0}+K_{0}{\left(1-e^{-\rho_{0}t}\right)}^{}\left\|\int_{0}^{t}\Psi \left(\tau ,t\right)d\tau \right\|\underset{0\leq \tau \leq t}{\sup}\left\|\tilde{A}\left(\tau \right)\right\|}$$

*if the assumptions 1 and 4 of Lemma A2 hold:*

(A13) $${\left\|F\left(t\right)\right\|}^{-1}\geq \varepsilon_{{}_{3Fm}}\left(t\right)=\frac{1}{1+\left\|\int_{0}^{t}e^{A_{0}\left(t-\tau \right)}\Psi \left(\tau ,t\right)d\tau \right\|\underset{0\leq \tau \leq t}{\sup}\left\|\tilde{A}\left(\tau \right)\right\|}$$

*if the assumptions 1 and 3 of Lemma A2 hold:*

(A14) $${\left\|F\left(t\right)\right\|}^{-1}\geq \varepsilon_{{}_{3Fm}}\left(t\right)=\frac{1}{1+\left\|\int_{0}^{t}e^{A_{0}\left(t-\tau \right)}\Psi \left(\tau ,t\right)d\tau \right\|\underset{0\leq \tau \leq t}{\sup}\left\|\tilde{A}\left(\tau \right)\right\|}$$

*if the assumptions 1 and 2 of Lemma A2 hold.*

**Proof.** It is a direct joint consequence of Banach´s Perturbation lemma and Lemma A2. Note that if M is a square real matrix and $\left\|E\right\|<1/\left\|M^{-1}\right\|$ then M+E is non-singular and

(A15) $$\left\|\;{\left(M+E\right)}^{-1}\right\|\leq \frac{\left\|M^{-1}\right\|}{1-\left\|M^{-1}\right\|\left\|E\right\|}$$

(Banach´s Perturbation lemma). Assume that $\left\|\;{\left(M+E\right)}^{-1}\right\|>\frac{\left\|M^{-1}\right\|}{1-\left\|M^{-1}\right\|\left\|E\right\|}$. Thus, the following contradiction follows:

(A16) $$1-\left\|M^{-1}\right\|\left\|E\right\|>1+\left\|M^{-1}\right\|\left\|E\right\|$$

Then, $\left\|\;{\left(M+E\right)}^{-1}\right\|\geq \frac{\left\|M^{-1}\right\|}{1+\left\|M^{-1}\right\|\left\|E\right\|}$. Thus, (A11) to (A14) follow from $F\left(t\right)=I_{n}-\int_{0}^{t}e^{A_{0}\left(t-\tau \right)}\tilde{A}\left(\tau \right)\Psi \left(\tau ,t\right)d\tau$,

(A17) $$\frac{\left\|M^{-1}\right\|}{1-\left\|M^{-1}\right\|\left\|E\right\|}\geq \left\|\;{\left(M+E\right)}^{-1}\right\|\geq \frac{\left\|M^{-1}\right\|}{1+\left\|M^{-1}\right\|\left\|E\right\|}$$

and the proof of Lemma A1, with the replacements $M\to I_{n}$, $E\to -\int_{0}^{t}e^{A_{0}\left(t-\tau \right)}\tilde{A}\left(\tau \right)\Psi \left(\tau ,t\right)d\tau$. □

**Lemma** **A3.**
*Assume that:*
*1)*$A_{0}$*is a stability matrix so that*$\left\|e^{A_{0}t}\right\|\leq K_{0}e^{-\rho_{0}t}$*;*$\forall t\in R_{0+}$*for some real constants*$K_{0}\geq 1$*and*$\rho_{0}>0$, *2)*$\underset{0\leq t<\infty }{\sup}\left\|\tilde{A}\left(t\right)\right\|=\varepsilon_{A}$*for*$\varepsilon_{A}\in {\left[0\;,\;\varepsilon_{A}^{*}\right]}^{}$*and some*$\varepsilon_{A}^{*}\in \left[0\;,\;1\right)$, *3)*$\left\|\Psi \left(t,\tau \right)\right\|\leq K_{\Psi }e^{-\rho_{\Psi }\left(t-\tau \right)}$*;*∀t(≥τ), τ∈R*with*$K_{\Psi }\geq 1$. 
*Then, the following properties hold:*

***(i)*** $\left\|F\left(t\right)\right\|\leq 1+\varepsilon_{A}^{*}\frac{K_{0}K_{\Psi }}{\rho_{0}-\rho_{\Psi }}\left(1-e^{-\left(\rho_{0}-\rho_{\Psi }\right)\;t}\right)$ *;* $\forall t\in R_{0+}$

*if* $\rho_{\Psi }<\rho_{0}$ *and*

$\left\|F\left(t\right)\right\|\geq 1-\varepsilon_{A}^{*}\frac{K_{0}K_{\Psi }}{\rho_{0}-\rho_{\Psi }}\left(1-e^{-\left(\rho_{0}-\rho_{\Psi }\right)\;t}\right)$ *;* $\forall t\in R_{0+}$ *if, furthermore,* $\varepsilon_{A}^{*}<\frac{\rho_{0}-\rho_{\Psi }}{K_{0}K_{\Psi }{\left(1-e^{-\left(\rho_{0}-\rho_{\Psi }\right)\;t}\right)}^{}}$

***(ii)*** $\left\|F\left(t\right)\right\|\leq 1+\varepsilon_{A}^{*}\frac{K_{0}K_{\Psi }}{\rho_{0}\rho_{\Psi }}\left(1-e^{-\rho_{0}\;t}\right)$ *;* $\forall t\in R_{0+}$ *, and*

$\left\|F\left(t\right)\right\|\geq 1-\varepsilon_{A}^{*}\frac{K_{0}K_{\Psi }}{\rho_{0}\rho_{\Psi }}\left(1-e^{-\rho_{0}\;t}\right)$ *;* $\forall t\in R_{0+}$ *if, furthermore,* $\varepsilon_{A}^{*}<\frac{\rho_{0}\rho_{\Psi }}{K_{0}K_{\Psi }{\left(1-e^{-\rho_{0}\;t}\right)}^{}}$

*Assume, in addition, that*

*4) The pair* $\left(A_{0}\;,\;b_{0}\right)$ *is controllable,*

*5)* $\underset{0\leq t<\infty }{\sup}\left\|\tilde{b}\left(t\right)\right\|=\varepsilon_{b}$ *for* $\varepsilon_{b}\in {\left[0\;,\;\varepsilon_{b}^{*}\right]}^{}$ *and some* $\varepsilon_{b}^{*}\in \left[0\;,\;1\right)$ *.*

*Then,* G(t*) *defined in (15) is subject to:*

***(iii)***

(A18) $$\left\|G\left(t*\right)\right\|\leq \varepsilon_{G}\left(t*\right)=\varepsilon_{G}^{*}=(\frac{K_{0}^{2}}{2\rho_{0}}\;\left(1-e^{-2\rho_{0}t*}\right)\left\|b_{0}\right\|\varepsilon_{b}^{*}+K_{0}^{2}K_{\Psi }\;\left\|b_{0}\right\|\varepsilon_{b}^{*}\varepsilon_{A}^{*}\;\times e^{-2\rho_{0}t*}\frac{e^{-2\rho_{\Psi }t*}+1-e^{\left(\rho_{0}-\rho_{\Psi }\right)t*}-e^{-\left(\rho_{0}+\rho_{\Psi }\right)t*}}{\rho_{0}^{2}-\rho_{\Psi }^{2}})\;\left\|G_{c\left[0,t*\right]}^{-1}\left(A_{0}\;,b_{0}\right)\right\|$$

**Proof.** Note that from Theorem 1 that $\rho_{\Psi }+\underset{0\leq t<+\infty }{\sup}\left\|\tilde{A}\left(t\right)\right\|\geq \rho_{0}>\rho_{\Psi }$ if $\rho_{\Psi }<\rho_{0}$ so that by direct calculations taking into account $\left\|e^{A_{0}t}\right\|\leq K_{0}e^{-\rho_{0}t}$, $\left\|\Psi \left(t,\tau \right)\right\|\leq K_{\Psi }e^{-\rho_{\Psi }\left(t-\tau \right)}$ and $\underset{0\leq t<\infty }{\sup}\left\|\tilde{A}\left(t\right)\right\|\leq \varepsilon_{A}^{*}$ $\left\|F\left(t\right)\right\|=\left\|I_{n}-\int_{0}^{t}e^{A_{0}\left(t-\tau \right)}\tilde{A}\left(\tau \right)\Psi \left(\tau ,t\right)d\tau \right\|\leq 1+\varepsilon_{A}^{*}\frac{K_{0}K_{\Psi }}{\rho_{0}-\rho_{\Psi }}\left(1-e^{-\left(\rho_{0}-\rho_{\Psi }\right)\;t}\right)$; $\forall t\in R_{0+}$ and $\left\|F\left(t\right)\right\|\geq 1-\varepsilon_{A}^{*}\frac{K_{0}K_{\Psi }}{\rho_{0}-\rho_{\Psi }}\left(1-e^{-\left(\rho_{0}-\rho_{\Psi }\right)\;t}\right)$ if

(A19) $$\varepsilon_{A}^{*}<\frac{\rho_{0}-\rho_{\Psi }}{K_{0}K_{\Psi }{\left(1-e^{-\left(\rho_{0}-\rho_{\Psi }\right)\;t}\right)}^{}}$$

Property (i) is proved. Property (ii) follows by applying H$\ddot{o}$lder´s inequality to $\int_{0}^{t}\left\|e^{A_{0}\left(t-\tau \right)}\right\|d\tau$ and $\int_{0}^{t}\left\|\Psi \left(\tau ,t\right)\right\|d\tau$ of the integral leading to the formula:

(A20) $$\left\|F\left(t\right)\right\|\leq 1+\underset{0\leq t<+\infty }{\sup}\left\|\;A\left(t\right)\right\|\int_{0}^{t}\left\|e^{A_{0}\left(t-\tau \right)}\right\|\left\|\Psi \left(\tau ,t\right)\right\|d\tau \;\leq 1+\underset{0\leq t<+\infty }{\sup}\left\|\;A\left(t\right)\right\|\left(\int_{0}^{t}{\left\|e^{A_{0}\left(t-\tau \right)}d\tau \right\|}^{2}\right){}^{1/2}\left(\int_{0}^{t}{\left\|\Psi \left(\tau ,t\right)\right\|}^{2}d\tau \right){}^{1/2}\;;\;\forall t\in R_{0+}$$

and, similarly, it follows that:

$$\left\|F\left(t\right)\right\|\geq 1-\underset{0\leq t<+\infty }{\sup}\left\|\;A\left(t\right)\right\|\int_{0}^{t}\left\|e^{A_{0}\left(t-\tau \right)}\right\|\left\|\Psi \left(\tau ,t\right)\right\|d\tau ;\forall t\in R_{0+}.$$

On the other hand, note that the conditions 1-5 allow to write the following chain of inequalities from (23)–(24):

(A21) $$\left\|G\left(t*\right)\right\|\leq \left(\left\|\int_{0}^{t*}e^{A_{0}\left(t*-\tau \right)}\tilde{b}\left(\tau \right)b_{0}^{T}e^{A_{0}^{T}\left(t*-\tau \right)}d\tau \right\|+\left\|\int_{0}^{t*}\int_{0}^{t*}e^{A_{0}\left(t*-\tau \right)}\tilde{A}\left(\tau \right)\Psi \left(\tau ,\sigma \right)b\left(\sigma \right)b_{0}^{T}e^{A_{0}^{T}\left(t*-\sigma \right)}d\sigma d\tau \right\|\right)G_{c\left[0,t*\right]}^{-1}\left(A_{0}\;,b_{0}\right)\;\leq \left(\frac{K_{0}^{2}}{2\rho_{0}}\;\left(1-e^{-2\rho_{0}t*}\right)\left\|b_{0}\right\|\underset{0\leq \tau \leq t*}{\sup}\left\|\tilde{b}\left(\tau \right)\right\|+K_{0}^{2}K_{\Psi }\;\left\|b_{0}\right\|\underset{0\leq \tau \leq t*}{\sup}\left\|\tilde{b}\left(\tau \right)\right\|\underset{0\leq \tau \leq t*}{\sup}\left\|\tilde{A}\left(\tau \right)\right\|\left(\int_{0}^{t*}\int_{0}^{t*}e^{-2\rho_{0}t+\left(\rho_{0}-\rho_{\Psi }\right)\tau +\left(\rho_{0}+\rho_{\Psi }\right)\sigma }d\sigma d\tau \right)\right)\;\times \left\|G_{c\left[0,t*\right]}^{-1}\left(A_{0}\;,b_{0}\right)\right\|\;=\left(\frac{K_{0}^{2}}{2\rho_{0}}\;\left(1-e^{-2\rho_{0}t*}\right)\left\|b_{0}\right\|\underset{0\leq \tau \leq t*}{\sup}\left\|\tilde{b}\left(\tau \right)\right\|+K_{0}^{2}K_{\Psi }\;\left\|b_{0}\right\|\underset{0\leq \tau \leq t*}{\sup}\left\|\tilde{b}\left(\tau \right)\right\|\underset{0\leq \tau \leq t*}{\sup}\left\|\tilde{A}\left(\tau \right)\right\|\;\left(\int_{0}^{t*}e^{-2\rho_{0}t+\left(\rho_{0}-\rho_{\Psi }\right)\tau }d\tau \int_{0}^{t*}e^{-\left(\rho_{0}+\rho_{\Psi }\right)\sigma }d\sigma \right)\right)\;\times \left\|G_{c\left[0,t*\right]}^{-1}\left(A_{0}\;,b_{0}\right)\right\|\;=(\frac{K_{0}^{2}}{2\rho_{0}}\;\left(1-e^{-2\rho_{0}t*}\right)\left\|b_{0}\right\|\underset{0\leq \tau \leq t*}{\sup}\left\|\tilde{b}\left(\tau \right)\right\|+K_{0}^{2}K_{\Psi }\;\left\|b_{0}\right\|\underset{0\leq \tau \leq t*}{\sup}\left\|\tilde{b}\left(\tau \right)\right\|\underset{0\leq \tau \leq t*}{\sup}\left\|\tilde{A}\left(\tau \right)\right\|\;\times e^{-2\rho_{0}t*}\frac{\left(e^{\left(\rho_{0}+\rho_{\Psi }\right)t}-1\right)\left(e^{-\left(\rho_{0}+\rho_{\Psi }\right)t}-1\right)}{\rho_{0}^{2}-\rho_{\Psi }^{2}})\left\|G_{c\left[0,t*\right]}^{-1}\left(A_{0}\;,b_{0}\right)\right\|$$

and Property (iii) is proved. □

**Remark** **A2.** *Note that the technical results for*F(t)*of Equations A1–A3 are valid for any*$t\in R_{0+}$*under the given sufficiency-type conditions rather that for the particular prefixed*t**chosen for generating the control law (12). However, the results for*G*depend explicitly on the control interval*[0,t*]*so that they are applicable for*t=t*.

## Appendix B. Guaranteed Reachability of the Current Linearized System from the Controllability of Its Nominal Counterpart

**Theorem** **A1.**
*Assume that the pair* $\left(A_{0}\;,\;b_{0}\right)$ *is controllable and* $\underset{0\leq t\leq t^{*}}{\sup}{\left\|\tilde{A}\left(t\right)\right\|}_{2}\leq \varepsilon_{A2}$ *and* $\underset{0\leq t\leq t^{*}}{\sup}\left\|\tilde{b}\left(t\right)\right\|\leq \varepsilon_{b2}$ *for sufficiently small real positive constants* $\varepsilon_{A2}=\varepsilon_{A2}\left(t^{*}\right)$ *and* $\varepsilon_{b2}=\varepsilon_{b2}\left(t^{*}\right)$ *depending on* $t^{*}$ *. Then, the following properties hold:*
***(i)***$G_{c\left[0,t*\right]}\left(A\left(t\right)\;,b\left(t\right)\right)$*is non-singular so that the current system is*$\mathrm{PR}\;\left(x^{*},t^{*}\;,x_{0}\right)$.
***(ii)*** *If* $\varepsilon_{A2}=\lambda_{A}\varepsilon_{0}\leq 1$ *and* $\varepsilon_{b2}=\lambda_{b}\varepsilon_{0}\leq 1$ *for some non-negative real constants* $\lambda_{A}$ *,* $\lambda_{b}$ *and* $\varepsilon_{0}\in \left[0\;,\;1\right)$ *then Property (i) is guaranteed if:*

(A22) $$\varepsilon_{0}<\frac{2\left|\rho_{0}\right|}{K_{0}^{2}}\frac{\lambda_{\min}\left(G_{c0\left[0,t*\right]}\left(A_{0},b_{0}\right)\right)}{\left(\;\left[4{\left\|b_{0}\right\|}_{2}^{2}\;+\left(2\left\|b_{0}\right\|+1\right)\lambda_{b}\right]\lambda_{A}\;+\left(2\left\|b_{0}\right\|+1\right)\lambda_{b}\left(1+\lambda_{A}\right)\right)\;\left|\;1-e^{-2\rho_{0}t^{*}}\right|}$$

**Proof.** Note from (13) and (36) that the controllability gramian of the current linearized system $G_{c\left[0,t*\right]}\left(A\left(t\right)\;,b\left(t\right)\right)$ is kept non-singular if that of its linearized counterpart $G_{c0\left[0,t*\right]}\left(A_{0}\;,b_{0}\right)$ is non-singular for all $t^{*}>0$ and, furthermore, ${\tilde{G}}_{c0\left[0,t*\right]}\left(A_{0}\;,b_{0}\right)=G_{c\left[0,t*\right]}\left(A\left(t\right),b\left(t\right)\right)-G_{c0\left[0,t*\right]}\left(A_{0}\;,b_{0}\right)$ has a sufficiently small norm such that, for any given matrix norm, the incremental controllability gramian satisfies $\left\|{\tilde{G}}_{c0\left[0,t*\right]}\left(A_{0}\;,b_{0}\right)\right\|<1/\left\|G_{c\left[0,t*\right]}^{-1}\left(A_{0},b_{0}\right)\right\|$ since:

(A23) $$G_{c\left[0,t*\right]}\left(A\left(t\right)\;,b\left(t\right)\right)=G_{c0\left[0,t*\right]}\left(A_{0}\;,b_{0}\right)+{\tilde{G}}_{c0\left[0,t*\right]}\left(A_{0}\;,b_{0}\right)\;=G_{c0\left[0,t*\right]}\left(A_{0}\;,b_{0}\right)\left(I_{n}+G_{c0\left[0,t*\right]}^{-1}\left(A_{0}\;,b_{0}\right){\tilde{G}}_{c0\left[0,t*\right]}\left(A_{0}\;,b_{0}\right)\right)$$

what follows from Banach´s Perturbation Lemma which ensures that $G_{c\left[0,t*\right]}\left(A\left(t\right)\;,b\left(t\right)\right)$ is non-singular, then, the current linearized system is $\mathrm{PR}\;\left(x^{*},t^{*}\;,x_{0}\right)$. Define $\Psi_{0}\left(t\;,\;\tau \right)=e^{A_{0}\left(t-\tau \right)}$, $\tilde{\Psi }\left(t,\;\tau \right)=\Psi \left(t,\;\tau \right)-\Psi_{0}\left(t\;,\;\tau \right)$; $\forall \tau \;,t\in {\left[0\;,\;t^{*}\right]}^{}\in R_{0+}$ and $\left({\tilde{\Delta }}_{c0\left[0,t\right]}\left(A_{0}\;,b_{0}\right)\right)\left(\tau \right)$ being the integrand defining ${\tilde{G}}_{c0\left[0,t\right]}\left(A_{0}\;,b_{0}\right)=\int_{0}^{t}\left({\tilde{\Delta }}_{c0\left[0,t\right]}\left(A_{0}\;,b_{0}\right)\right)\;\left(\tau \right)d\tau$; $\forall t\in {\left[0\;,\;t^{*}\right]}^{}\in R_{0+}$. Simple direct calculations to expand in additive terms the incremental controllability gramian yield ${\tilde{G}}_{c0\left[0,t\right]}\left(A_{0}\;,b_{0}\right)={\int_{0}^{t^{*}}\tilde{\Delta }}_{c0\left[0,\tau \right]}\left(A_{0}\;,b_{0}\right)d\tau$ with:

(A24) $$\left\|\left({\tilde{\Delta }}_{c0\left[0,t\right]}\left(A_{0}\;,b_{0}\right)\right)\left(\tau \right)\right\|\leq 2\left\|\Psi_{0}\left(t\;,\;\tau \right)b_{0}b_{0}^{T}\tilde{\Psi }\left(t\;,\;\tau \right)\right\|+\left\|\tilde{\Psi }\left(t\;,\;\tau \right)b_{0}b_{0}^{T}\tilde{\Psi }{}_{T}\left(t\;,\;\tau \right)\right\|\;+\left\|\Psi_{0}\left(t\;,\;\tau \right)\left(b_{0}{\tilde{b}}^{T}+\tilde{b}b_{0}+\tilde{b}{\tilde{b}}^{T}\right)\Psi_{0}^{T}\left(t\;,\;\tau \right)\right\|+\left\|\tilde{\Psi }\left(t\;,\;\tau \right)\left(b_{0}{\tilde{b}}^{T}+\tilde{b}b_{0}+\tilde{b}{\tilde{b}}^{T}\right){\tilde{\Psi }}^{T}\left(t\;,\;\tau \right)\right\|\;+2\left\|\tilde{\Psi }{\left(t\;,\;\tau \right)}^{}\left(b_{0}{\tilde{b}}^{T}+\tilde{b}b_{0}+\tilde{b}{\tilde{b}}^{T}\right)\Psi_{0}^{T}\left(t\;,\;\tau \right)\right\|$$

Note that:

$${\left\|\Psi_{0}\left(t^{*}\;,\;\tau \right)\right\|}_{2}\leq K_{0}e^{-\rho_{0}(t^{*}-\tau ),}{\int_{0}^{t^{*}}\left\|\Psi_{0}\left(t^{*}\;,\;\tau \right)\right\|}_{2}d\tau \leq \left(K_{0}/\left|\rho_{0}\right|\right)|1-e^{-\rho_{0}t^{*}}|,$$

$${\int_{0}^{t^{*}}\left\|\Psi_{0}\left(t^{*}\;,\;\tau \right)\right\|}_{2}^{2}d\tau \leq \left(K_{0}^{2}/2\left|\rho_{0}\right|\right)\left|1-e^{-2\rho_{0}t^{*}}\right|$$

and, since $\underset{0\leq t\leq t^{*}}{\sup}{\left\|\tilde{A}\left(t\right)\right\|}_{2}\leq \varepsilon_{A2}$, one has $\int_{0}^{t^{*}}{\left\|\tilde{\Psi }\left(t^{*}\;,\;\tau \right)\;\right\|}_{2}^{2}d\tau \leq \left(K_{0}^{2}\varepsilon_{A}^{2}/2\left|\rho_{0}\right|\right)\left|1-e^{-2\rho_{0}t^{*}}\right|$ and, since furthermore $\underset{0\leq t\leq t^{*}}{\sup}\left\|\tilde{b}\left(t\right)\right\|\leq \varepsilon_{b2}$, one gets from (A23) and (A24) that:

(A25) $${\left\|{\tilde{G}}_{c0\left[0,t^{*}\right]}\left(A_{0}\;,b_{0}\right)\right\|}_{2}=\left\|\int_{0}^{t^{*}}\left({\tilde{\Delta }}_{c0\left[0,t\right]}\left(A_{0}\;,b_{0}\right)\right)\;\left(\tau \right)d\tau \right\|{}_{2}\;\leq \frac{K_{0}^{2}}{2\left|\rho_{0}\right|}\left(\varepsilon_{A2}\left[2{\left\|b_{0}\right\|}_{2}^{2}\;\left(1+\varepsilon_{A2}\right)+\left(2\left\|b_{0}\right\|+\varepsilon_{b2}\right)\varepsilon_{b2}\right]\;+\left(2\left\|b_{0}\right\|+\varepsilon_{b2}\right)\varepsilon_{b2}\left(1+\varepsilon_{A2}^{2}\right)\right)\;\left|\;1-e^{-2\rho_{0}t^{*}}\right|$$

(A26) $$=\frac{K_{0}^{2}{\left\|b_{0}\right\|}_{2}}{\left|\rho_{0}\right|}\left(\varepsilon_{A2}{\left\|b_{0}\right\|}_{2}+\varepsilon_{b2}\;\right)\;\left|\;1-e^{-2\rho_{0}t^{*}}\right|+o\left(\varepsilon_{A}\right)+o\left(\varepsilon_{b}\right)$$

Then, if $\frac{K_{0}^{2}{\left\|b_{0}\right\|}_{2}}{\left|\rho_{0}\right|}\left(\varepsilon_{A2}{\left\|b_{0}\right\|}_{2}+\varepsilon_{b2}\;\right)\;\left|\;1-e^{-2\rho_{0}t^{*}}\right|<1/\left\|G_{c\left[0,t*\right]}^{-1}\left(A_{0},b_{0}\right)\right\|$ and, if $\max\left(\varepsilon_{A2},\;\varepsilon_{b2}\right)$ is small enough for the given $t^{*}$ then there exists a positive real constant $\varepsilon =\varepsilon \left(\varepsilon_{A2}\;,\;\varepsilon_{b2},\;t^{*}\right)$ satisfying:

$$\varepsilon \leq 1/\left\|G_{c\left[0,t*\right]}^{-1}\left(A_{0},b_{0}\right)\right\|-\frac{K_{0}^{2}{\left\|b_{0}\right\|}_{2}}{\left|\rho_{0}\right|}\left(\varepsilon_{A2}{\left\|b_{0}\right\|}_{2}+\varepsilon_{b2}\;\right)\;\left|\;1-e^{-2\rho_{0}t^{*}}\right|$$

such that ${\left\|{\tilde{G}}_{c0\left[0,t*\right]}\left(A_{0}\;,b_{0}\right)\right\|}_{2}<\lambda_{\min}\left(G_{c0\left[0,t*\right]}\left(A_{0},b_{0}\right)\right)=1/{\left\|G_{c0\left[0,t*\right]}^{-1}\left(A_{0},b_{0}\right)\right\|}_{2}$. This implies that if $G_{c0\left[0,t*\right]}\left(A_{0}\;,b_{0}\right)$ is non-singular then $G_{c\left[0,t*\right]}\left(A\left(t\right)\;,b\left(t\right)\right)$ is non-singular and the first part of the proof is complete. On the other hand, if $\varepsilon_{A2}=\lambda_{A}\varepsilon_{0}\leq 1$ and $\varepsilon_{b2}=\lambda_{b}\varepsilon_{0}\leq 1$ for some real constant $\varepsilon_{0}\in \left[0\;,\;1\right)$, [4], then $\varepsilon_{A2}^{2}=\lambda_{A}^{2}\varepsilon_{0}^{2}\leq \varepsilon_{A2}=\lambda_{A}\varepsilon_{0}\leq 1$ and $\varepsilon_{b2}^{2}=\lambda_{b}^{2}\varepsilon_{0}^{2}\leq \varepsilon_{b2}=\lambda_{b}\varepsilon_{0}\leq 1$ so that one has from (A25) that:

(A27) $${\left\|{\tilde{G}}_{c0\left[0,t^{*}\right]}\left(A_{0}\;,b_{0}\right)\right\|}_{2}\;\leq \frac{K_{0}^{2}}{2\left|\rho_{0}\right|}\left(\;\left[4{\left\|b_{0}\right\|}_{2}^{2}\;+\left(2\left\|b_{0}\right\|+1\right)\varepsilon_{b2}\right]\varepsilon_{A2}\;+\left(2\left\|b_{0}\right\|+1\right)\varepsilon_{b2}\left(1+\varepsilon_{A2}\right)\right)\;\left|\;1-e^{-2\rho_{0}t^{*}}\right|\;\leq \frac{\varepsilon_{0}K_{0}^{2}}{2\left|\rho_{0}\right|}\left(\;\left[4{\left\|b_{0}\right\|}_{2}^{2}\;+\left(2\left\|b_{0}\right\|+1\right)\lambda_{b}\right]\lambda_{A}\;+\left(2\left\|b_{0}\right\|+1\right)\lambda_{b}\left(1+\lambda_{A}\right)\right)\;\left|\;1-e^{-2\rho_{0}t^{*}}\right|$$

and ${\left\|{\tilde{G}}_{c0\left[0,t*\right]}\left(A_{0}\;,b_{0}\right)\right\|}_{2}<1/{\left\|G_{c0\left[0,t*\right]}^{-1}\left(A_{0},b_{0}\right)\right\|}_{2}$ holds from (A27) if (A22) holds. The proof is complete. □
